# Zmynd11 is essential for neurogenesis by coordinating H3K36me3 modification of Epha2 and PI3K signaling pathway

**DOI:** 10.1186/s13578-025-01392-z

**Published:** 2025-04-25

**Authors:** Xu Yang, Lan Li, Wenzheng Qu, Xuejun Cheng, Jinyu Zhang, Yan Sun, Suxiao Liu, Guoping Peng, Rui Zheng, Xuekun Li

**Affiliations:** 1 Children’s Hospital, Zhejiang University School of Medicine, National Clinical Research Center for Child Health, Hangzhou, China; 2 The Institute of Translational Medicine, Zhejiang University School of Medicine, Hangzhou, China; 3 Binjiang Institute of Zhejiang University, Hangzhou, China; 4 Department of Neurology, the First Affiliated Hospital, Zhejiang University School of Medicine, Hangzhou, China; 5 Center for Reproductive Medicine, Department of Obstetrics, Zhejiang Provincial People’s Hospital, Hangzhou, China

**Keywords:** 10p15.3 deletion syndrome, Zmynd11, Neurogenesis, H3K36me3 modification, Epha2, PI3K

## Abstract

10p15.3 deletion syndrome is caused by the deficiency of MYND-type zinc finger domain-containing protein 11 (*ZMYND11*) and featured by global developmental delay, intellectual disability, behavioral abnormalities, etc. Although the roles of Zmynd11 is intensively studied in cancer, the function and associated mechanisms of Zmynd11 in neurodevelopment remain largely unknown. Here, we show that Zmynd11 displays abundant and dynamic expression pattern during embryonic neurodevelopment. *Zmynd11* deficiency impairs embryonic neurogenesis and neurodevelopment in vitro and in vivo, and inhibits morphological maturation of neurons. Mechanistically, *Zmynd11* deficiency leads to decreased Epha2 and disrupts PI3K signaling pathway. Under *Zmynd11* deficient condition, H3K36me3 modification on *Epha2* promoter abnormally increases and the binding of RNA polymerase II decreases. The restoration of PI3K signaling pathway by exogenous Epha2 can rescue aberrant neurogenesis induced by *Zmynd11* depletion in vitro and in vivo. Collectively, our study reveals the essential function of Zmynd11 in neurogenesis via coordinating H3K36me3 modification of *Epha2* and PI3K signaling pathway.

**Supplementary Information:**

The online version contains supplementary material available at 10.1186/s13578-025-01392-z.

## Introduction

MYND-type zinc finger domain-containing protein 11 (Zmynd11) primarily represses gene expression by specifically recognizing histone modifications H3.3K36me3 [1–4]. Previous studies have shown that ZMYND11 plays critical function as tumor suppressor [3, 5–9], and decreased ZMYND11 promotes tumor cell growth and migration, which is correlated with poor outcomes and lower survival rate [6–9]. In addition, ZMYND11 was remarkably decreased in glioblastoma multiform (GBM) tissues, while exogenous Zmynd11 significantly reduced tumor cell proliferation and invasion, and promoted cell cycle arrest and apoptosis [10]. In pediatric high-grade gliomas (pHGGs), ZMYND11 binding to H3.3-G34R maintained the expression of key neural progenitor genes and promoted tumorigenesis [6, 11]. A recent study showed that Zmynd11 exhibits tumor repressive function by interacting with arginine methyltransferase PRMT5 and prohibiting the formation of HNRNPA1-mediated stress granule [9]. Collectively, these studies have uncovered multifaced function and associated mechanisms of Zmynd11 in tumor.

ZMYND11 is highly expressed in fetal brain [12, 13], and *ZMYND11* haploinsufficiency causes 10p15.3 deletion syndrome in human, which is featured by global developmental delay, intellectual disability, behavioral abnormalities, etc. [14–19]. In a cellular model, *Zmynd11* deficiency impaired neuronal generation through regulating brain-specific isoform splicing [20]. However, the exact function and associated mechanisms of Zmynd11 in regulating neurodevelopment remain underexplored.

Here, we show that Zmynd11 displays abundant and dynamic expression pattern during embryonic neurodevelopment. *Zmynd11* deficiency inhibits proliferation and induces aberrant neuronal differentiation of embryonic neuronal progenitor cells (eNPCs) in vitro and in vivo, and impairs morphological maturation of neurons. Mechanistically, *Zmynd11* deficiency leads to decreased Epha2 and disrupts PI3K signaling pathway. Under *Zmynd11* deficiency condition, H3K36me3 modification at *Epha2* promoter abnormally increases and the binding of RNA polymerase II decreases. The restoration of PI3K signaling pathway by exogenous Epha2 can rescue aberrant neurogenesis induced by *Zmynd11* depletion in vitro and in vivo. Collectively, our study reveals the essential function of Zmynd11 in neurogenesis and neurodevelopment through regulating PI3K signaling pathway.

## Results

### *Zmynd11* deficiency inhibits the proliferation and induces aberrant neuronal differentiation of NPCs

To determine the roles of Zmynd11 in neurodevelopment, we first examined its expression pattern in the brain of mice. Immunofluorescence staining of brain sections collected from embryonic day 14 (E14), E17 and postnatal day 0 (P0) mice revealed that Zmynd11 signal was colocalized with embryonic neural progenitor cells (eNPCs) marker Nestin and neuronal marker β-III tubulin (Tuj1) (Figure S1A). The intensity of Zmynd11 signal was increased from E14 to P0 (Figure S1A). In consistency, Western blot (WB) and qRT-PCR assays showed that the level of Zmynd11 was increased during neurodevelopment (Figure S1B-1D).

Next, we isolated eNPCs and neurons from embryonic mice brains (E14), and astrocytes from the brains of new born pups (P0). Immunostaining, WB and qRT-PCR assays results showed that the expression of Zmynd11 was remarkably elevated upon the differentiation of eNPCs (Figure S1E-1H). Notably, Map2^+^ Neurons and Gfap^+^ astrocytes exhibited the greater level of Zmynd11 compared to that of Nestin^+^ eNPCs (Figure S1I-1L). Collectively, these results suggest that Zmynd11 displays a dynamic expression pattern during neurodevelopment.

**Fig. 1:**
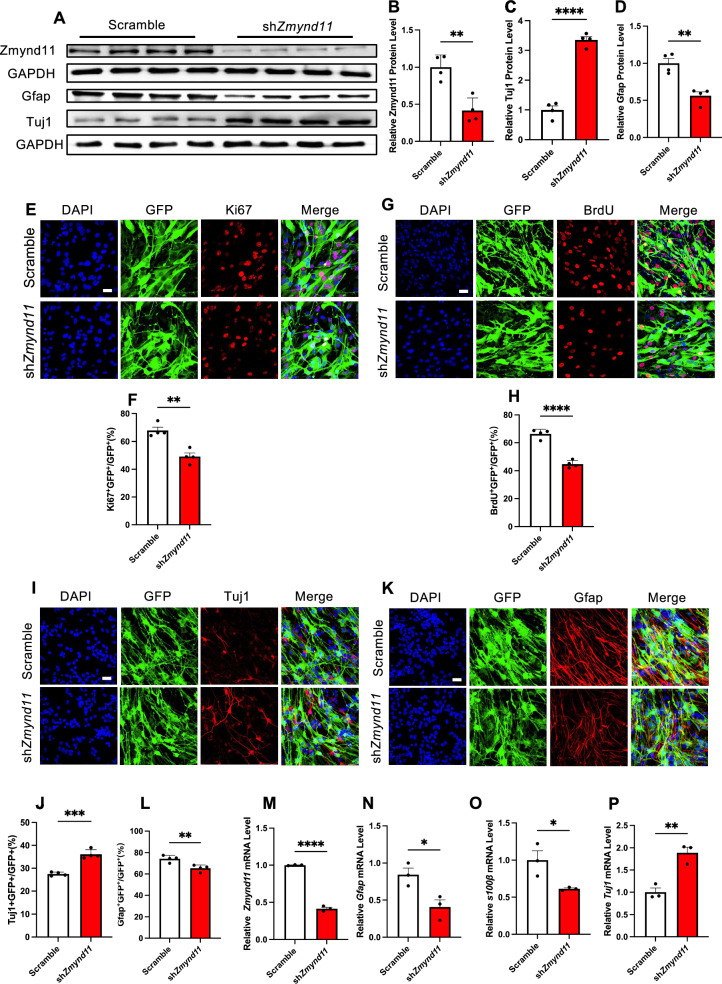
*Zmynd11* depletion impairs the proliferation and differentiation of eNPCs in vitro. **A**–**D** Representative Western blot (WB) images of Zmynd11, neuronal marker Tuj1 and astrocytic marker Gfap (**A**). *Zmynd11* KD significantly reduced the level Zmynd11 protein in proliferating eNPCs (**B**). Under differentiation condition, *Zmynd11* KD significantly reduced Gfap (**C**), but increased Tuj1 protein compared to scramble group (Ctrl) (**D**). n = 3 biologically independent experiments for each group. Data represent mean ± SEM, unpaired t test; *, p < 0.05; **, p < 0.01; ***, p < 0.001; n.s. no significance. **E** Representative images of Ki67 immunofluorescence staining with eNPCs infected with lentivirus expressing scramble and shRNA against *Zmynd11* (sh*Zmynd11*) compared to scramble group (Ctrl). eNPCs were infected with lentivirus for 72 h. Scale bar, 50 μm. **F** Quantification results showed that *Zmynd11* knockdown (KD) led to a decreased percentage of Ki67^+^ cells. n = 4 biologically independent experiments for each group. Data represent mean ± SEM, unpaired t test; *, p < 0.05; **, p < 0.01; ***, p < 0.001; n.s. no significance. **G** Representative images of BrdU immunofluorescence staining with eNPCs infected with lentivirus expressing scramble and sh*Zmynd11*. eNPCs were infected with lentivirus for 72 h, and BrdU was incorporated for 6 h at a final concentration of 5 μM. Scale bar, 50 μm. **H** Quantification results showed that *Zmynd11* KD reduced the percentage of BrdU^+^ cells. n = 4 biologically independent experiments for each group. Data represent mean ± SEM, unpaired t test; *, p < 0.05; **, p < 0.01; ***, p < 0.001; n.s. no significance. **I** Representative images of Tuj1 immunofluorescence staining with eNPCs infected with lenti-scramble and lenti-sh*Zmynd11*. After eNPCs were infected with lentivirus for 72 h, eNPCs differentiation was induced for 48 h. Scale bar, 50 μm. **J** Quantification results showed that *Zmynd11* KD enhanced the percentage of Tuj1^+^ cells. n = 4 biologically independent experiments for each group. Data represent mean ± SEM, unpaired t test; *, p < 0.05; **, p < 0.01; ***, p < 0.001; n.s. no significance. **K** Representative images of Gfap immunofluorescence staining with eNPCs infected with lenti-scramble and lenti-sh*Zmynd11*. After eNPCs were infected with lentivirus for 72 h, eNPCs differentiation was induced for 48 h. Scale bar, 50 μm. **L** Quantification results showed that *Zmynd11* KD reduced the percentage of Gfap^+^ cells. n = 4 biologically independent experiments for each group. Data represent mean ± SEM, unpaired t test; *, p < 0.05; **, p < 0.01; ***, p < 0.001; n.s. no significance. **M**–**P** qRT-PCR assay results showed that *Zmynd11* KD significantly reduced the mRNA levels of *Zmynd11* (**M**), *Gfap* (**N**), s100β (**O**) and increased the mRNA level of *Tuj1* (**P**). n = 3 independent experiments for each group. Data represent mean ± SEM, unpaired t test; *, p < 0.05; **, p < 0.01; ***, p < 0.001; n.s. no significance

To examine the effects of Zmynd11 on neurogenesis, we performed *Zmynd11* knockdown (KD) with lentivirus expressing short hairpin RNA against *Zmynd11* in eNPCs. *Zmynd11* KD significantly reduced protein levels of Zmynd11 in proliferating eNPCs (Fig. 1A, B). Upon the differentiation of eNPCs, *Zmynd11* KD significantly reduced the level of actrocytic marker Gfap, but enhanced the level of neuronal marker Tuj1 (Fig. 1A, C, D). Immunostaining and quantification results showed that the percentages of Ki67^+^GFP^+^/GFP^+^ and BrdU^+^GFP^+^/GFP^+^ cells were significantly decreased in *Zmynd11* KD group compared to scramble group (Fig. 1E–H). Upon the differentiation of eNPCs, the percentage of Tuj1^+^GFP^+^/GFP^+^ was significantly increased (Fig. 1I, J), but the percentage of Gfap^+^GFP^+^/GFP^+^ cells was significantly decreased in *Zmynd11* KD group compared to scramble group (Fig. 1K, L). qRT-PCR assay results also showed that *Zmynd11*KD significantly reduced *Gfap* expression, but increased *Tuj1* expression (Fig. 1M–P).

**Fig. 2:**
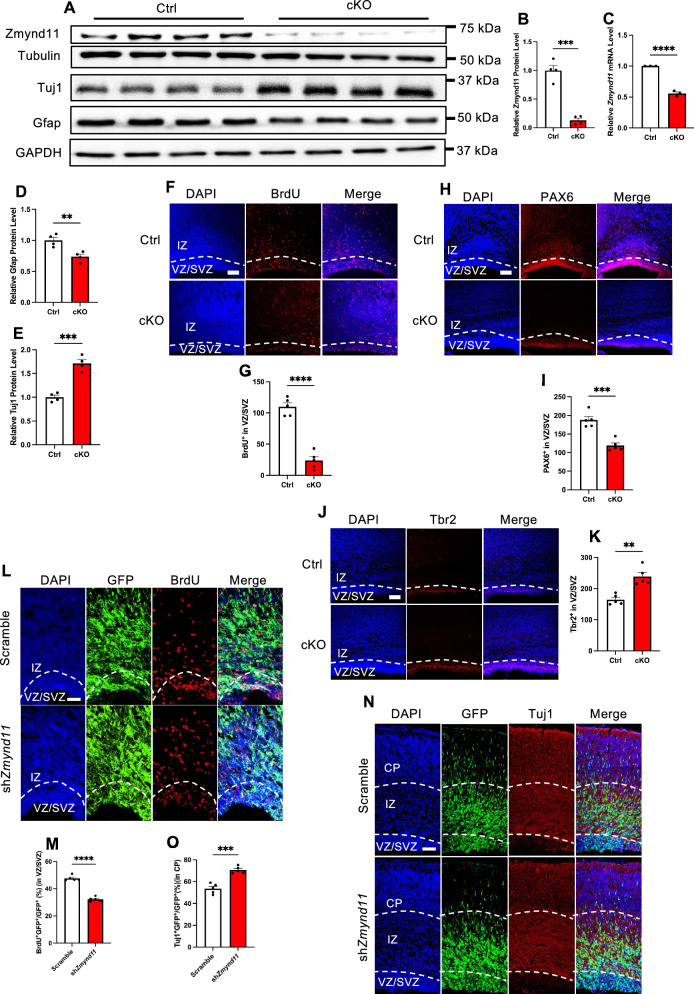
*Zmynd11* deficiency disrupts neurogenesis in embryonic brain. **A** Representative WB images of Zmynd11, Tuj1 and Gfap in the brain of *Zmynd11*^*loxp/loxp*^ (Ctrl) and *Nestin*-Cre;*Zmynd11*^*−/−*^ (cKO) pups at the age of postnatal day 0 (P0). **B**, **C** Quantification results showed that cKO mice had decreased protein and mRNA levels of Zmynd11. n = 4 independent experiments for each group in (**B**) and n = 3 biologically independent experiments for each group in (**C**). Data represent mean ± SEM, unpaired t test; *, p < 0.05; **, p < 0.01; ***, p < 0.001; n.s. no significance. **D**, **E** Quantification results showed that cKO mice had decreased Gfap (**D**), but increased Tuj1 (**E**). n = 4 independent experiments for each group. Data represent mean ± SEM, unpaired t test; *, p < 0.05; **, p < 0.01; ***, p < 0.001; n.s. no significance. **F** Representative images of BrdU immunostaining with brain sections of Ctrl and cKO mice (P0). BrdU was injected (100 mg/kg, intraperitoneally [i.p.]) 1.5 h prior to the sacrifice. Scale bar, 50 μm. **G** Quantification results showed that the number of BrdU^+^ cells was significantly decreased in VZ/SVZ of cKO mice compared to Ctrl. n = 5 pups for each group. Data are presented as mean ± SEM; *p < 0.05; **p < 0.01; ***p < 0.001, ****p < 0.0001, unpaired Student’s t test. **H** Representative images of radial glial marker PAX6 immunostaining with brain sections of Ctrl and cKO mice at the age of P0. Scale bar, 50 μm. **I** Quantification results showed that the number of PAX6^+^ cells in VZ/SVZ was significantly decreased in VZ/SVZ of cKO mice compared to Ctrl. n = 5 pups for each group. Data are presented as mean ± SEM; *p < 0.05, **p < 0.01; ***p < 0.001, unpaired Student’s t test. **J** Representative images of intermediate progenitor cell marker Tbr2 immunostaining with brain sections of Ctrl and cKO mice (P0). Scale bar, 50 μm. **K** Quantification results showed the number of Tbr2^+^ cells was significantly increased in VZ/SVZ of cKO mice compared to Ctrl. n = 5 pups for each group. Data are presented as mean ± SEM; *p < 0.05, **p < 0.01, unpaired Student’s t test. **L** Representative images of BrdU immunostaining with scramble and *Zmynd11* KD brain sections. Scramble and sh*Zmynd11* plasmids were delivered into E14 fetal brain via in utero electroporation, and animals were sacrificed for assay at E17. BrdU was injected (100 mg/kg, i.p.) 1.5 h prior to the sacrifice. Scale bar, 50 μm. **M** Quantification results showed the percentage of BrdU^+^GFP^+^/GFP^+^ in VZ/SVZ was significantly decreased in *Zmynd11* KD group compared to Ctrl. n = 5 fetuses at least from 4 pregnant mice for each group. Data are presented as mean ± SEM; *p < 0.05; **p < 0.01; ***p < 0.001, ****p < 0.0001, unpaired Student’s t test. **N** Representative images of Tuj1 immunostaining with scramble and *Zmynd11* KD brain sections as in (**L**). Scale bar, 50 μm. **O** Quantification results showed the percentage of Tuj1^+^GFP^+^/GFP^+^ was significantly increased in cortical plate (CP) layer of *Zmynd11* KD group compared to Ctrl. n = 5 fetuses at least from 4 pregnant mice for each group. Data are presented as mean ± SEM; *p < 0.05; **p < 0.01; ***p < 0.001, ****p < 0.0001, unpaired Student’s t test

Next, we generated conditional knockout (cKO) mice (*Zmynd11*^loxp/loxp^; *Nestin*-Cre) by crossing *Zmynd11*^loxp/loxp^ mice with *Nestin*-Cre mice (Figure S2 A). cKO pups (P0) displayed less body weight compared to Control (Ctrl) group (*Zmynd11*^loxp/loxp^) (Figures S2B, S2 C). WB assay and qRT-PCR results showed that the protein and mRNA levels of Zmynd11 was significantly decreased in the brain of cKO pups (P0) (Fig. 2A–C). In consistency with in vitro study, the protein level of Gfap was significantly decreased, but the level of Tuj1 was significantly increased in the brain of cKO pups compared to Ctrl (P0) (Fig. 2D, E). Ctrl and cKO pups were administrated with 5-bromo-2’-deoxyuridine (BrdU), and immunofluorescence staining showed that cKO mice displayed remarkable decrease of BrdU^+^ cells in ventricular zone/subventricular zone (VZ/SVZ) regions compared to that of Ctrl group (Fig. 1F, G). The number of PAX6^+^ radial glial cells (RGCs), one type of NPC, was also significantly decreased in VZ/SVZ of cKO mice (Fig. 1H, I). Of note, the number of Tbr2^+^ cells, one type of neuronal progenitor cells, was significantly increased in VZ/SVZ of cKO mice compared to Ctrl (Fig. 1J, K).

Next, we adopted in utero electroporation (IUE) to deliver scramble and sh*Zmynd11* plasmids into the brain of E14 embryonic fetus, respectively, which were analyzed on E17. Immunostaining showed that the percentages of BrdU^+^GFP^+^/GFP^+^ and Pax6^+^GFP^+^/GFP^+^ cells were significantly decreased in VZ/SVZ of *Zmynd11* KD group compared to scramble group (Fig. 2L, M, S2D, S2E). The percentages of Tuj1^+^GFP^+^/GFP^+^ cells and Tbr2^+^GFP^+^/GFP^+^ cells were significantly increased in VZ/SVZ of *Zmynd11* KD group compared to scramble group (Fig. 2N, O, S2F, S2G). *Zmynd11* KD also disrupted the migration of new born neurons, indicated by the decreased percentage of GFP^+^ cells in VZ/SVZ and cortical plate (CP), and increased percentage of GFP^+^ cells in intermediate zone (IZ) (Figures S2H-S2K). Collectively, these results suggest that *Zmynd11* deficiency leads to abnormal neurogenesis in vitro and in vivo.

### *Zmynd11* depletion inhibits the morphological development of neurons

**Fig. 3:**
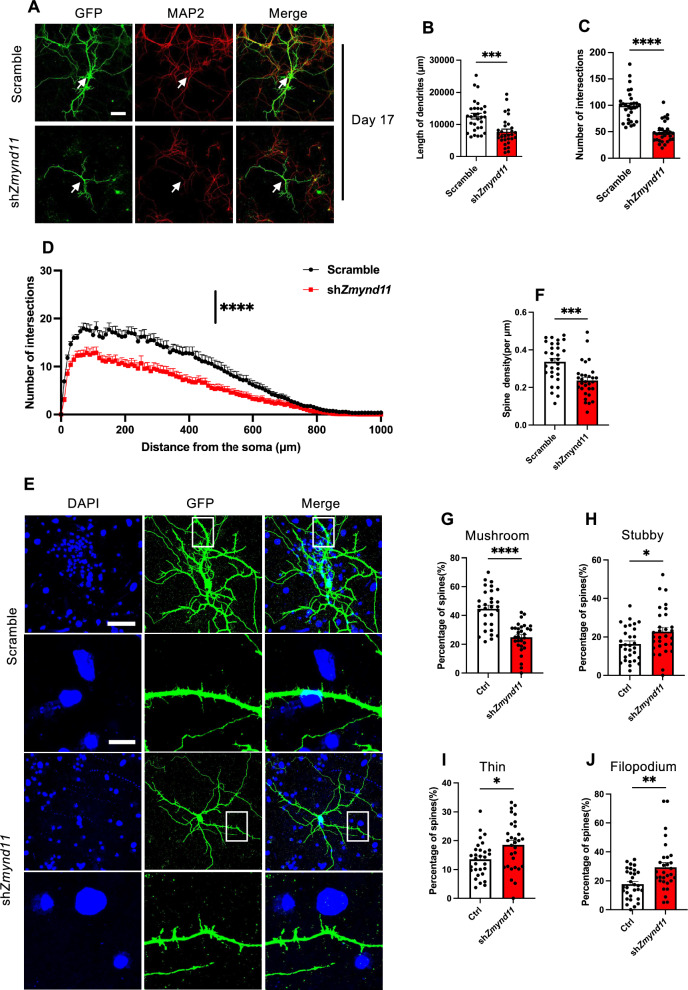
*Zmynd11* deficiency inhibits morphological development of neurons. **A** Representative images of cultured cortical neurons at DIV 17. Primary neurons were isolated from the cortical tissues of E14 fetal brains. Scramble and sh*Zmynd11* plasmids were transfected at DIV 14, and cells were collected for assay at DIV 17. Scale bar, 50 μm. **B**, **C** Quantification results showed that *Zmynd11* KD significantly reduced the dendritic length (**B**) and the number of branch points (**C**) of cortical neurons compared to scramble group. n = 30 cells were analyzed for each group. Data are presented as mean ± SEM; *p < 0.05, **p < 0.01, ***p < 0.001, ****p < 0.0001, unpaired Student’s t test. **D** Sholl analysis results showed that *Zmynd11* KD reduced the dendritic complexity of cortical neurons compared to scramble group. n = 30 cells were analyzed for each group. Data are presented as mean ± SEM; *p < 0.05, **p < 0.01, ***p < 0.001, ****p < 0.0001, unpaired Student’s t test. **E** Representative images of neurons cultured for 21 days in vitro. Scramble and sh*Zmynd11* plasmids were transfected at DIV 18, and cells were collected for assays at DIV 21. Scale bar, 5 μm. **F**–**J** Quantification results showed that *Zmynd11* KD reduced the spine density (**F**) and the proportion of mushroom spines (**G**), but increased the proportion of stubby (**H**), thin (**I**), filopodium (**J**) spines. n = 30 cells were analyzed for each group. Data are presented as mean ± SEM; *p < 0.05, **p < 0.01, ***p < 0.001, ****p < 0.0001; unpaired Student’s t test

Next, we isolated primary neurons from cortical tissues of embryonic brain (E14), and performed *Zmynd11* KD assay at days in vitro (DIV) 14 and DIV18, and analyzed at DIV 17 and DIV 21, respectively. We observed that *Zmynd11* KD reduced the length of dendrites, number of intersections at DIV 17 (Fig. 3A–C). Sholl analysis showed that *Zmynd11* KD led to decreased complexity of neurons (Fig. 3D). In addition, at DIV 21, neurons exhibited fewer spine density in *Zmynd11* KD group (Fig. 3E, F). *Zmynd11* KD also led to reduced mushroom spines, and increased stubby spines, thin spines, filopodia spines (Fig. 3E, G–J). Collectively, these results suggest that *Zmynd11* depletion impairs morphological development of neurons.

### *Zmynd11* depletion leads to the dysregulated PI3K signaling pathway

**Fig. 4:**
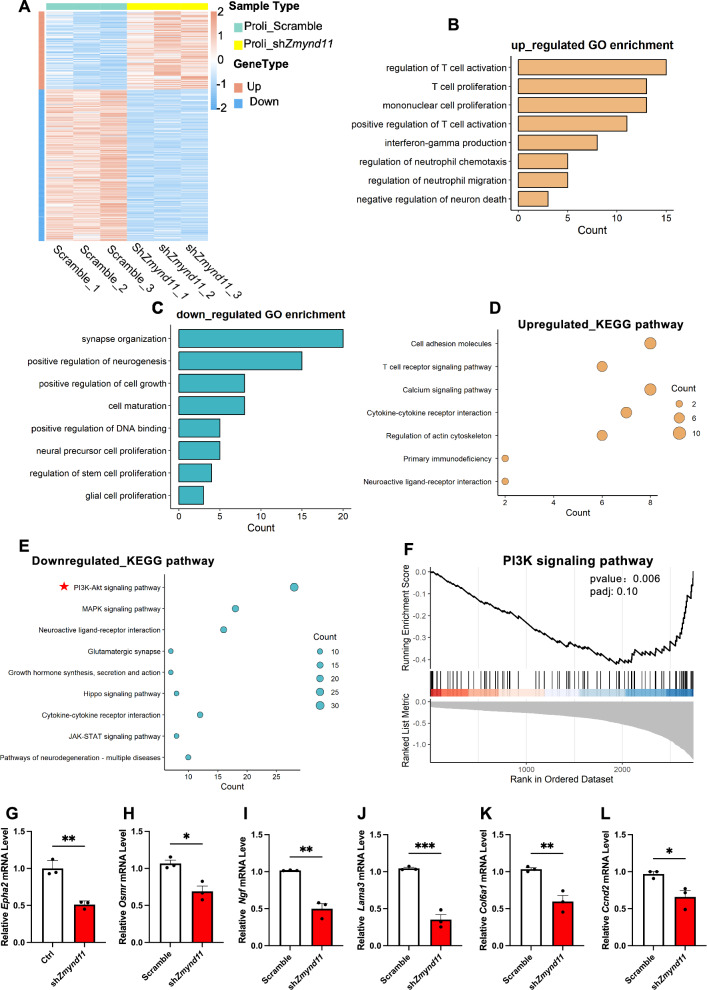

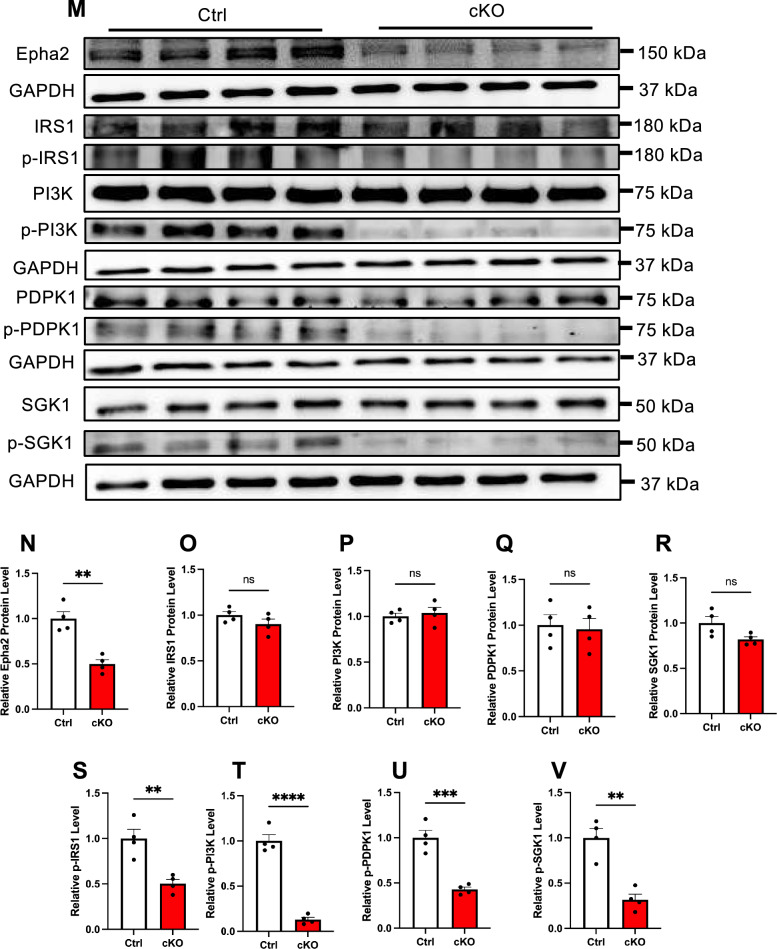
*Zmynd11* deficiency alters the transcriptome and disrupts PI3K signaling pathway. **A** Heatmap shows differentially expressed genes (DEGs) between scramble and *Zmynd11* KD eNPCs. **B** Gene ontology (GO) analysis showed that upregulated genes were highly enriched for the terms related with T cell activation and proliferation in *Zmynd11* KD eNPCs. **C** GO analysis showed that downregulated genes were highly enriched for the terms related with neuronal development in *Zmynd11* KD eNPCs. **D** KEGG analysis showed the upregulated pathways in *Zmynd11* KD eNPCs. **E** KEGG analysis showed the downregulated pathways including PI3K signaling pathway in *Zmynd11* KD eNPCs. **F** Gene Set Enrichment Analysis (GSEA) map of influenced genes in PI3K signaling pathway. **G**–**L** qRT-PCR assay results showed that *Zmynd11* KD significantly reduced the mRNA level of *Epha2* (**G**), *Osmr* (**H**), *Ngf* (**I**), *Lama3* (**J**), *Col6a1* (**K**), *Ccnd2* (**L**), key components of PI3K signaling pathway. n = 3 biologically independent experiments. Data are presented as mean ± SEM; *p < 0.05, **p < 0.01, ***p < 0.001, unpaired Student’s t-test. **M** Representative WB images of Zmynd11, Epha2, IRS1, p-IRS1, PI3K, p-PI3K, PDPK1, p-PDPK1, SGK1, p-SGK1 in the cortical tissues of Ctrl and cKO mice. Tubulin and GAPDH were used as internal controls. **N**–**V** Quantification results showed that level of Epha2 was significantly decreased in the brain of cKO mice compared to Ctrl (**N**). The levels of total IRS1 (**O**), PI3K (**P**), PDPK1 (**Q**), and SGK1 (**R**) showed no difference between Ctrl and cKO mice, but the levels of p-IRS1 (**S**), p-PI3K (**T**), p-PDPK1 (**U**), and p-SGK1 (**V**) were remarkably decreased in cKO mice compared to Ctrl. n = 4 biologically independent experiments. Data are presented as mean ± SEM; *p < 0.05, **p < 0.01, ***p < 0.001, ****p < 0.0001, unpaired Student’s t test

To uncover the molecular mechanism of *Zmynd11* in regulating neurogenesis, we performed RNA sequencing (RNA-seq) with eNPCs infected with lentivirus-scramble and lenti-sh*Zmynd11*, respectively. RNA-seq data analysis identified 536 differentially expressed genes (DEGs), 355 genes downregulated and 181 genes upregulated, in *Zmynd11* KD group (Fig. 4A, Table S1). Gene Ontology (GO) enrichment analysis of DEGs showed that the upregulated genes were significantly enriched in terms including T cell activation and proliferation, etc., while downregulated DEGs showed a high enrichment in neurogenesis, neural precursor/stem cell proliferation, etc. (Fig. 4B, C). In addition, the Kyoto Encyclopedia of Genes and Genomes (KEGG) analysis revealed that the upregulated DEGs were implicated in pathways associated with T cell signaling and cytokine interaction, etc. (Fig. 4D). Notably, the downregulated DEGs were implicated in pathways associated with the phosphoinositide 3-kinase (PI3K) signaling pathway, mitogen-activated protein kinase (MAPK) signaling pathway, etc. (Fig. 4E). Gene set enrichment analysis (GSEA) further showed that *Zmynd11* KD significantly downregulated the expression of genes involved in PI3K signaling pathway (Fig. 4F). qRT-PCR assay results showed that *Zmynd11* KD *bona fide* resulted in the decreased expression of genes associated with PI3K signaling pathway including *Epha2*, *Osmr*, *Ngf*, *Lama3*, *Col6a1*, *Ccnd2*, etc. (Fig. 4G–L, S3A-S3I). WB assay results showed that *Zmynd11* KD significantly reduced the protein levels of total Epha2 (Figure S3J, S3K). Although the protein levels of total IRS1, PI3K, PDPK1 and SGK1 showed no difference between Ctrl and *Zmynd11* KD groups, the levels of phosphorylated-IRS1 (p-IRS1), phosphorylated-PI3K (p-PI3K), phosphorylated-PDPK1 (p-PDPK1) and phosphorylated-SGK1 (p-SGK1) was significantly decreased in *Zmynd11* KD NPCs compared to Ctrl group (Figure S3J, S3L-S3S).

Furthermore, we examined PI3K pathway components with cortical brain tissues of Ctrl and cKO mice (P0) and found that *Zmynd11* deficiency resulted in significant reduction of total Epha2, p-IRS1, p-PI3K, p-PDPK1 and p-SGK1, but not altered the protein levels of total IRS1, PI3K, PDPK1 and SGK1 (Fig. 4M–V), which was consistent with in vitro results. Together, these results suggest that *Zmynd11* depletion leads to the dysregulation of PI3K signaling pathway.

### *Zmynd11* regulates Epha2 expression through recognizing H3K36me3 modification

**Fig. 5:**
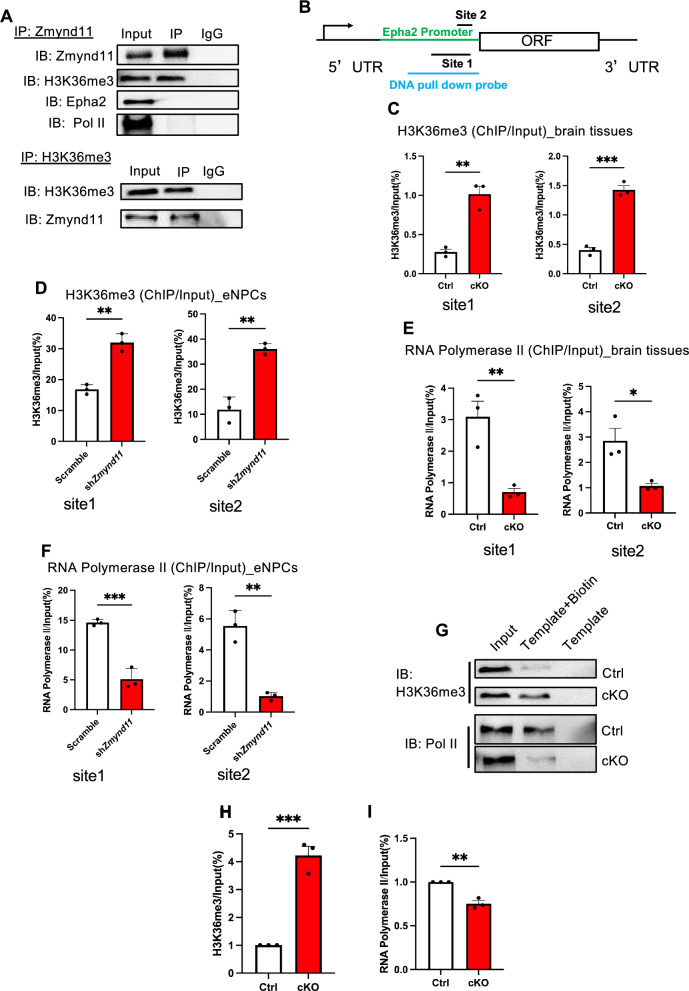
*Zmynd11* deficiency results in abnormal *Epha2* transcription in vivo. **A** Immunoprecipitation (IP) followed by WB assay showed that Zmynd11 easily precipitated H3K36me3, and vice versa. Zmynd11 did not directly interact with Epha2 and RNA Polymerase II. **B** Schematic illustration of primers design for analyzing *Epha2* promoter and DNA pull down assay. **C** Chromatin immunoprecipitation-qPCR (ChIP-qPCR) and quantification results showed that the enrichment of H3K36me3 at *Epha2* promotor was significantly increased in cKO brain tissues compared to Ctrl. n = 3 biologically independent experiments. Data are presented as mean ± SEM; *p < 0.05, **p < 0.01, ***p < 0.001, ****p < 0.0001, unpaired Student’s t test. **D** ChIP-qPCR and quantification results showed that the enrichment of H3K36me3 at *Epha2* promotor was significantly increased in *Zmynd11* KD eNPCs compared to Ctrl. n = 3 biologically independent experiments. Data are presented as mean ± SEM; *p < 0.05, **p < 0.01, ***p < 0.001, ****p < 0.0001, unpaired Student’s t test. **E** ChIP-qPCR and quantification results showed that the binding of RNA Polymerase II to *Epha2* promotor was significantly decreased in cKO brain tissues compared to Ctrl. n = 3 biologically independent experiments. Data are presented as mean ± SEM; *p < 0.05, **p < 0.01, ***p < 0.001, ****p < 0.0001, unpaired Student’s t test. **F** ChIP-qPCR and quantification results showed that the binding of RNA Polymerase II to *Epha2* promotor was significantly decreased in *Zmynd11* KD eNPCs compared to Ctrl. n = 3 biologically independent experiments. Data are presented as mean ± SEM; *p < 0.05, **p < 0.01, ***p < 0.001, unpaired Student’s t-test. **G**–**I** DNA pull down followed by WB assay and quantification results showed that the enrichment of H3K36me3 at *Epha2* promotor was significantly increased, but RNA Polymerase II binding was significantly decreased in cKO brain tissues compared to Ctrl. n = 3 biologically independent experiments. Data are presented as mean ± SEM; *p < 0.05, **p < 0.01, ***p < 0.001, unpaired Student’s t-test

Previous studies have shown that Zmynd11 regulates gene expression as an H3K36me3 reader [1–4, 21–24]. We then performed immunoprecipitation (IP) with cortical brain tissues of wild-type mice using Zmynd11 antibody followed by WB assays, and observed that Zmynd11 antibody easily precipitated H3K36me3, and vice versa (Fig. 5A), and similar results were observed in eNPCs (Figure S4 A). Of note, we did not observe a direct interaction between Zmynd11 and Epha2 or RNA polymerase II (Fig. 5A). ChIP-qPCR results showed that the enrichment of H3K36me3 modification was remarkably increased at the promoter region of *Epha2* in cortical brain tissues of cKO mice (Fig. 5B, C) and *Zmynd11* KD eNPCs (Figs. 5D, S4B) compared to Ctrl group, while the level of total H3K36me3 showed no difference between groups (Figures S4C-S4E). In addition, the binding of RNA polymerase II (Pol II) to *Epha2* promoter regions was significantly decreased in cortical brain tissues of cKO mice (Fig. 5E) and *Zmynd11* KD NPCs (Figs. 5F, S4F) compared to Ctrl groups, but the level of total Pol II showed no difference between groups (Figures S4G, S4H). DNA IP with a biotin probe targeting to *Epha2* promoter showed that the enrichment of H3K36me3 increased, but the enrichment of RNA Pol II decreased at *Epha2* promoter regions in cortical brain tissues of cKO mice compared to Ctrl group (Fig. 5G–I). Collectively, these results suggest that Zmynd11 regulates Epha2 expression through effecting H3K36me3 modification and RNA Pol II binding at *Epha2* promoter.

### Exogenous *Epha2* rescues neurodevelopmental deficits induced by *Zmynd11* deficiency

**Fig. 6:**
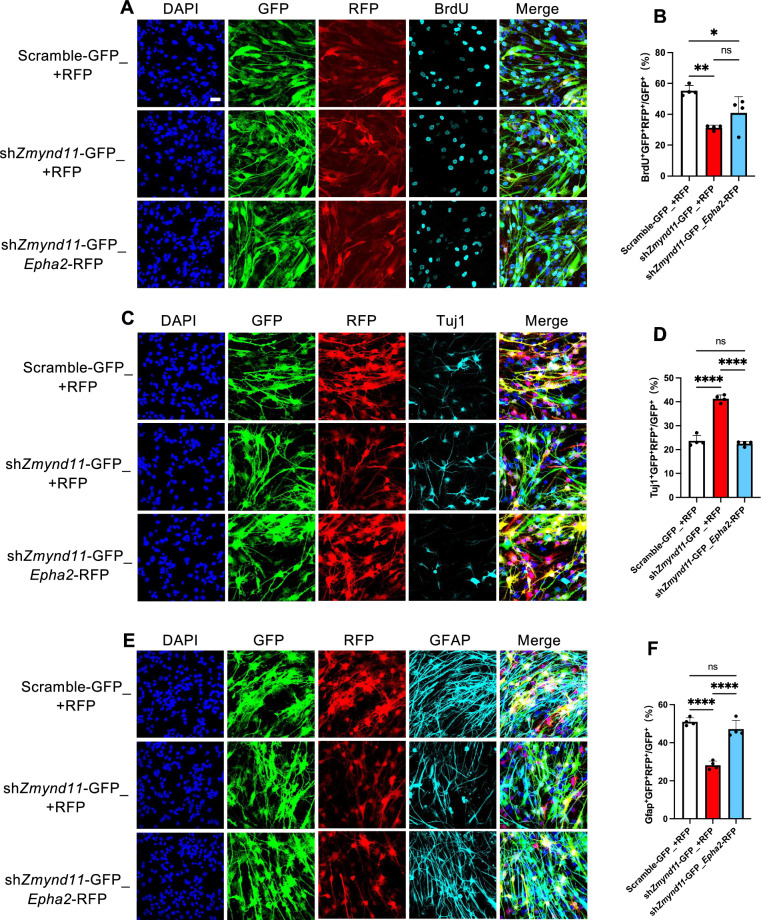

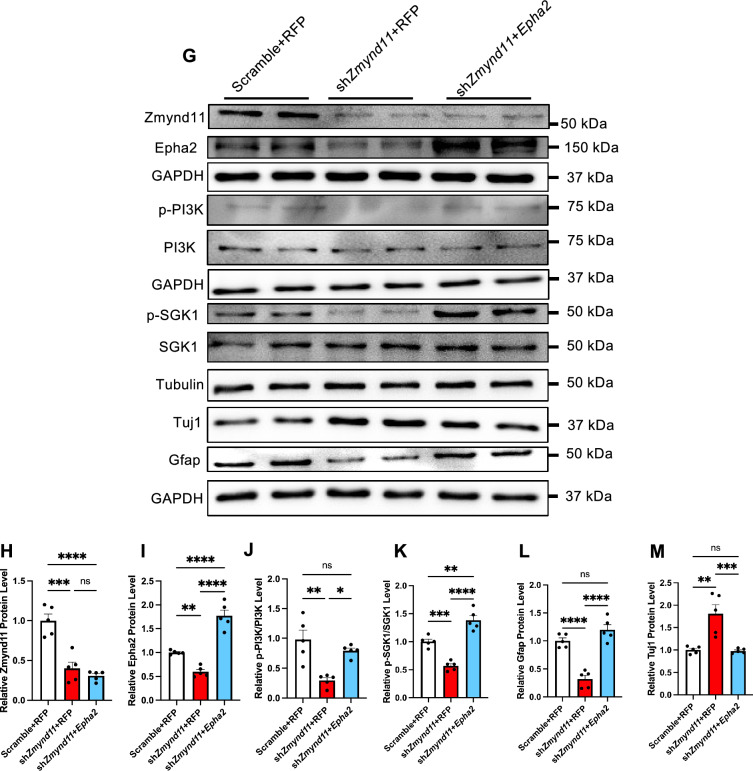
Exogenous *Epha2* rescues neurogenic deficits and restores PI3K signaling in *Zmynd11* KD eNPCs. **A** Representative images of BrdU immunostaining of WT eNPCs infected with lenti-scramble-GFP, lenti-sh*Zmynd11*-GFP, and lenti-sh*Zmynd11*-GFP + lenti-*Epha2*-RFP, respectively. BrdU was incorporated for 6 h at a final concentration of 5 μM. Scale bar, 50 μm. **B** Quantification results showed that ectopic Epha2 significantly enhanced the percentage of BrdU^+^ cells in *Zmynd11* KD eNPCs compared to Ctrl. n = 4 biologically independent experiments. Data are presented as mean ± SEM; *p < 0.05, **p < 0.01, ***p < 0.001, and ****p < 0.0001, one-way ANOVA analysis followed by Tukey’s multiple-comparison test, F_(2, 9)_ = 13.73. **C** Representative images of Tuj1 immunostaining of WT eNPCs infected with lenti-scramble-GFP, lenti-sh*Zmynd11*-GFP, and lenti-sh*Zmynd11*-GFP + lenti-*Epha2*-RFP, respectively. Scale bar, 50 μm. **D** Quantification results showed that ectopic Epha2 significantly reduced the percentage of Tuj1^+^ cells in *Zmynd11* KD group compared to Ctrl. n = 4 biologically independent experiments. Data are presented as mean ± SEM; *p < 0.05, **p < 0.01, ***p < 0.001, and ****p < 0.0001, one-way ANOVA analysis followed by Tukey’s multiple-comparison test, F_(2, 9)_ = 154.6. **E** Representative images of immunostaining of WT eNPCs infected with lenti-scramble-GFP, lenti-sh*Zmynd11*-GFP, and lenti-sh*Zmynd11*-GFP + lenti-*Epha2*-RFP, respectively. Scale bar, 50 μm. **F** Quantification results showed that ectopic Epha2 significantly enhanced the percentage of Gfap^+^ cells in *Zmynd11* KD group compared to Ctrl. n = 4 biologically independent experiments. Data are presented as mean ± SEM; *p < 0.05, **p < 0.01, ***p < 0.001, and ****p < 0.0001, one-way ANOVA analysis followed by Tukey’s multiple-comparison test, F_(2, 9)_ = 59.39. **G**–**M** Representative WB images (**G**) and quantification results showed that exogenous Epha2 did not affect the protein level of Zmynd11 (**H**), but significantly enhanced the level of Epha2 (**I**) in *Zmynd11* KD eNPCs compared to Ctrl. Exogenous Epha2 significantly enhanced p-PI3K/PI3K (**J**), p-SGK1/SGK1 (**K**), and Gfap (**L**), but reduced the level of Tuj1 (**M**) in *Zmynd11* KD eNPCs compared to Ctrl. n = 4 biologically independent experiments. Data are presented as mean ± SEM; *p < 0.05, **p < 0.01, ***p < 0.001, and ****p < 0.0001, one-way ANOVA analysis followed by Tukey’s multiple-comparison test, F_(2, 12)_ = 30.36 for (**H**), F _(2, 12)_ = 61.28 for (**I**), F _(2, 12)_ = 12.68 for (**J**), F _(2, 12)_ = 50.39 for (**K**), F _(2, 12)_ = 40.36 for (**L**), F _(2, 12)_ = 15.66 for (**M**)

Next, we aim to determine whether exogenous Epha2 could rescue neurodevelopmental deficits induced by *Zmynd11* deficiency. Exogenous *Epha2* was delivered into eNPCs under scramble and *Zmynd11* KD conditions, respectively. Under the proliferating condition, exogenous Epha2 restored the proliferating capability of eNPCs indicated by the increased percentage of BrdU^+^ cells in *Zmynd11* KD group (Fig. 6A, B). Under the differentiation condition, exogenous Epha2 reduced the percentage of Tuj1^+^ neurons, but increased the percentage of Gfap^+^ astrocytes in *Zmynd11* KD group (Fig. 6C–F). In addition, exogenous Epha2 restored morphological deficits of cultured neurons induced by *Zmynd11* KD (Figures S5A-S5D).

We next examined the effects of exogenous Epha2 on PI3K signaling pathway. qRT-PCR assay results showed that exogenous Epha2 significantly increased mRNA levels of *Epha2* and *Gfap*, but reduced *Tuj1* mRNA in *Zmynd11* KD eNPCs, while the level of *Zmynd11* mRNA was not changed (Figures S5E-S5H). WB assay results showed that exogenous Epha2 significantly restored the decreased levels of Epha2, p-PI3K, p-SGK1 in *Zmynd11* KD eNPCs (Fig. 6G–K). In addition, exogenous Epha2 significantly increased the level of Gfap protein, but reduced the level of Tuj1 protein in *Zmynd11* KD eNPCs (Fig. 6G, L, M).

**Fig. 7:**
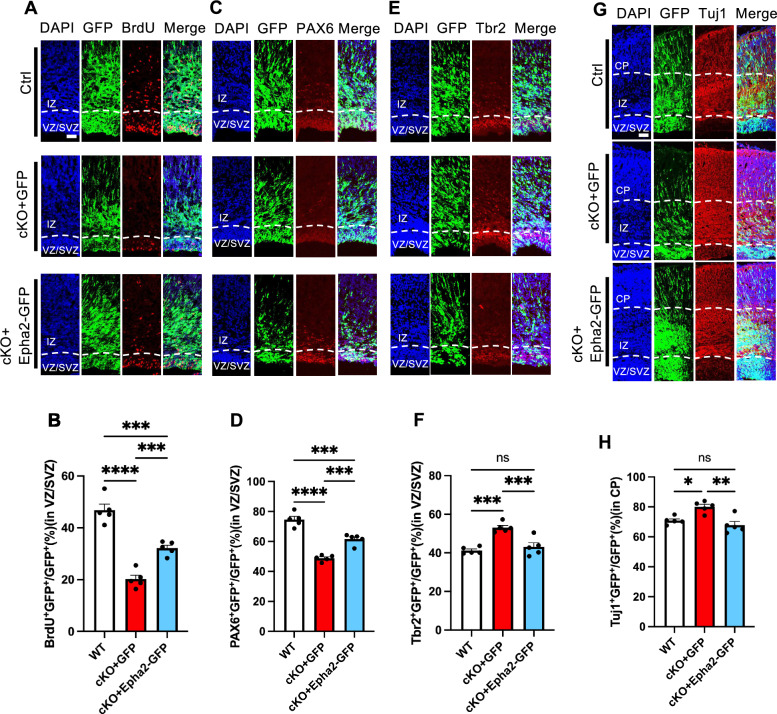
Exogenous *Epha2* rescues neurogenic deficits in vivo. **A** Representative images of BrdU immunostaining with Ctrl, cKO + GFP and cKO + Epha2-GFP brain sections. Venus GFP and Epha2 plasmids were delivered into fetal brain via in utero electroporation at E14, and animals were sacrificed for assay at E17. BrdU was injected (100 mg/kg, i.p.) 1.5 h prior to the sacrifice. Scale bar, 50 μm. **B** Quantification results showed the percentage of BrdU^+^GFP^+^/GFP^+^ in VZ/SVZ was significantly increased in cKO + Epha2-GFP group compared to cKO-GFP group. n = 5 fetuses at least from 4 pregnant mice for each group. Data are presented as mean ± SEM; *p < 0.05; **p < 0.01; ***p < 0.001, ****p < 0.0001, one-way ANOVA analysis followed by Tukey’s multiple-comparison test, F_(2, 12)_ = 59.67. **C** Representative images of PAX6 immunostaining with Ctrl, cKO + GFP and cKO + Epha2-GFP brain sections. Scale bar, 50 μm. **D** Quantification results showed the percentage of PAX6^+^GFP^+^/GFP^+^ in VZ/SVZ was significantly increased in cKO + Epha2-GFP group compared to cKO-GFP group. n = 5 fetuses at least from 4 pregnant mice for each group. Data are presented as mean ± SEM; *p < 0.05; **p < 0.01; ***p < 0.001, ****p < 0.0001, one-way ANOVA analysis followed by Tukey’s multiple-comparison test, F_(2,12)_ = 64.34. **E** Representative images of Tbr2 immunostaining with Ctrl, cKO + GFP and cKO + Epha2-GFP brain sections. Scale bar, 50 μm. **F** Quantification results showed the percentage of Tbr2^+^GFP^+^/GFP^+^ in VZ/SVZ was significantly decreased in cKO + Epha2-GFP group compared to cKO-GFP group. n = 5 fetuses at least from 4 pregnant mice for each group. Data are presented as mean ± SEM; *p < 0.05; **p < 0.01; ***p < 0.001, ****p < 0.0001, one-way ANOVA analysis followed by Tukey’s multiple-comparison test, F_(2, 12)_ = 19.6. **G** Representative images of Tuj1 immunostaining with Ctrl, cKO + GFP and cKO + Epha2-GFP brain sections. Scale bar, 50 μm. **H** Quantification results showed the percentage of Tuj1^+^GFP^+^/GFP.^+^ in VZ/SVZ was significantly decreased in cKO + Epha2-GFP group compared to cKO-GFP group. n = 5 fetuses at least from 4 pregnant mice for each group. Data are presented as mean ± SEM; *p < 0.05; **p < 0.01; ***p < 0.001, ****p < 0.0001, one-way ANOVA analysis followed by Tukey’s multiple-comparison test, F_(2, 12)_ = 11.81

Finally, we determined whether exogenous Epha2 could rescue neurodevelopmental deficits in vivo. *Epha2* was delivered into Ctrl and cKO E14 embryonic brains via IUE, respectively, and immunostaining assays were performed at E17. We observed that exogenous Epha2 significantly enhanced the percentages of BrdU^+^GFP^+^ cells and PAX6^+^GFP^+^ cells in VZ/SVZ of cKO fetal brains (Fig. 7A–D), but reduced the percentages of Tbr2^+^GFP^+^ in VZ/SVZ and Tuj1^+^GFP^+^ in CP of cKO fetal brains (Fig. 7E–H). Collectively, these results suggest that exogenous Epha2 could rescue neurodevelopmental deficits induced by *Zmynd11* deficiency.

## Discussion

Embryonic and postnatal neurodevelopment in mammalian is intensively regulated by diverse mechanisms, including environment, genetics and epigenetics [25–29]. In the present study, we have found that Zmynd11 widely presents in embryonic brain and shows increased expression along neurodevelopment. *Zmynd11* deficiency impairs the proliferating capability of neuronal progenitor cells, but aberrantly promotes production of neurons and inhibits glial differentiation. *Zmynd11* deficiency promotes the binding of SETD2, a methyltransferase for depositing H3K36me3 modification, and enhances H3K36me3 modification at *Epha2* promoter region, which consequently inhibits PI3K signaling pathway and leads to neurodevelopmental deficits. Exogenous Epha2 restores the inhibited PI3K signaling pathway and aberrant neurogenesis and neurodevelopment. Collectively, our study has revealed the important function of Zmynd11 in regulating neurogenesis and associated mechanisms.

*Zmynd11* haploinsufficiency induces 10p15.3 deletion syndrome in human, and patients display neurological deficits including intellectual disability, behavioral abnormalities, and seizures [14, 18, 19, 30–33]. However, the knowledge about underlying mechanism for these phenotypes are very limited. Ectopic *Zmynd11* and *Zmynd11* deficiency both impairs neurogenesis and neurodevelopment in vitro, suggesting a complicated function of Zmynd11 in brain [20, 34]. Our present data show that *Zmynd11* deficiency not only disturbs the proliferation of embryonic neural progenitor cells, but leads to aberrant neuronal production and prohibits neuronal maturation in vitro and in vivo. In addition, our data show that *Zmynd11* deficiency reduced Epha2, which led to the dysregulation of PI3K signaling pathway. The restoration of PI3K signaling pathway by ectopic Epha2 partially rescues neurodevelopmental deficits. PI3K signaling pathway is regulated by Epha2 [35–38], and is essential for neurogenesis and neurodevelopment [26, 39, 40]. Altogether, our study reveals the important function of Zmynd11 regulates neurodevelopment via Epha2-PI3K pathway.

Previous studies have shown that Zmynd11 recognizes H3K36me3 on H3.3 (H3.3K36 me3) modification and serves as a transcription repressor [1–4, 21–24]. However, some result also indicate that H3K36me3 inhibits gene expression [41–43]. Our data show that *Zmynd11* deficiency results in the increased expression of some genes while the expression of some genes was decreased in neuronal progenitor cells (NPCs), suggesting that Zmynd11 plays multifacet function in regulating gene expression during neurodevelopment. In addition, *Zmynd11* deficiency led to increased H3K36me3 modification at the *Epha2* promoter, while the total level of H3K36me3 modification showed no difference between Ctrl and cKO in vitro and in vivo. It would be intriguing to examine whether *Zmynd11* deficiency affected the binding of SETD2 to *Epha2* promoter region and associated mechanism. Collectively, these results suggest that the function of H3K36me3 on gene expression could be context dependent, i.e., where and when H3K36me3 mark is placed, and what reader protein binds to this modification and the additional surrounding marks leading to different biological outcomes.

Although it has been shown that Zmynd11 regulates RNA polymerase II elongation in tumor, we observed that *Zmynd11* deficiency reduced the binding of RNA Polymerase II at *Epha*2 promoter in eNPCs and brain samples. These data suggest that Zmynd11 maintains gene expression by effecting SETD2-catalyzed H3K36me3 and Pol II binding to specific genes in brain. Of note, considering that *Zmynd11* deficiency alters the expression of a set of genes including *Epha2*, further studies are requisite to uncover their function in neurodevelopment. Collectively, our study reveals a new layer how Zmynd11 regulates gene expression during neurodevelopment and provides novel insights for the mechanisms underlying the neurological deficits of 10p15.3 deletion syndrome in human.

## Materials and methods

### Animals

*Zmynd11*^loxp/loxp^ mice were obtained from GemPharmatech (Nanjing, China), and crossed with *Nestin*-Cre mice to generate conditional knockout mice (*Nestin*-Cre;*Zmynd11*^loxp/loxp^, cKO). Wild-type (WT) C57BL/6 pregnant mice were purchased from Shanghai SLAC Laboratory Animal Center, and gestational day 0.5 was determined by observation of vaginal plug. All animals were housed under a 12-h light/dark cycle at the Experimental Animal Center of Zhejiang University with free access to food and water. The mice were genotyped by PCR, and the sequences of used primers was included in Table S2.

### Isolation and culture of embryonic neuronal progenitor cells (eNPCs)

The isolation and culture of eNPCs were performed as described previously [44]. The cortical tissues of E14 fetal brains were dissected and cut into pieces followed by the digestion with 0.1% Trypsin (25200072, GIBCO) for 5 min at 37 °C. Samples were centrifuged at a speed of 1500 rpm, and pellets were collected and washed with DMEM/F-12 medium (D8437, Sigma). Cells were cultured with DMEM/F-12 medium containing 2% B27 (minus vitamin A) (17504044, Thermo Fisher Scientific), 20 ng/mL FGF2 (100-18B, Peprotech), 2 mM L-Glutamine (25030149, GIBCO), 20 ng/mL EGF (AF-100–15, Peprotech) and 1% antibiotic–antimycotic (15140-122, Thermo Fisher Scientific) in a 5% CO_2_ incubator at 37 °C.

For the proliferation and differentiation assays, eNPCs were plated onto the coverslips (354087, BD), and BrdU at a final concentration of 5 μM was added to the medium for 6 h. At the scheduled time point, cells were fixed with 4% PFA and immunofluorescence staining was performed. The antibody information was included in Table S3.

### Isolation and culture of primary neurons and astrocytes

Primary neuron were isolated from cortical tissues of E14 fetal brains as described previously [44]. Briefly, tissues were digested with 0.25% trypsin at 37°C for 30 min followed by a centrifuge at a speed of 1500 rpm. Around 1.5 × 10^5^ neurons were plated onto a slice (Corning) coated with poly-D-lysine (5 mg/mL, Sigma, P0899-10). Neurons were plated in the plating medium containing DMEM (Gibco, 11095-080), 10% FBS (Gibco, 10091-148), 1% L-Glutamine (Gibco, 5030-149), and 1% sodium pyruvate (Gibco, 11360-070). 4 h later, the medium was replaced with growing medium containing neurobasal (Gibco 21103-049), 2% B27 (Gibco 25030-149), 0.25% L-Glutamine and 0.125% Glutamax (Thermo 35050061).

For dendritic spine analysis was performed as described previously [45]. Spine numbers were counted on the entire dendritic segment and spine density per unit of dendritic segment length was calculated. The shape of each dendritic spine was analyzed by calculating the ratio of the width/the length of each spine. All experiments were from a minimum of three independent cultures and at least ten transfected neurons per slice. Dendritic spine subgroups classified on the basis of the following morphology types: stubby spines (< 0.5 µm in length, lacking a clear head), mushroom spines (mushroom-shaped head, approximately 1 µm in length), thin spines (with an elongated narrow neck with a distinctive head), and filopodia (longer than 5 µm in length).

Astrocytes were isolated as previous description [44]. In brief, cortical and hippocampal tissues of postnatal day 1 pups were dissected. The tissues samples were digested with 0.25% trypsin for 25 min at 37°C followed by a centrifuge at a speed of 1500 rpm. The pellets were resuspended and around 1 × 10^7^ cells were plated into T25 culture flask coated with DMEM medium supplemented with 10% FBS, 1% antibiotic–antimycotic, and 2 mM L-Glutamine. The medium was replaced every 2 days. Upon cell growth proportion reaching to 80% area of the T25 flask, the flasks were put on the shaker (240 rpm) overnight at 37°C, and cells were further cultured with flesh culture medium.

### BrdU labeling and immunostaining

For in vivo neurogenesis assay, pregnant mice and postnatal day 1 pups were injected with BrdU (B5002, Sigma) intraperitoneally (i.p.) at a dosage of (100 mg/kg body weight). 1.5 h later, animals were deeply anesthetized with isoflurane and transcardically perfused with cold PBS followed by cold 4% PFA. Fetal brains were removed and postfixed in 4% PFA overnight. After dehydrated with 30% sucrose at 4°C, brain samples were embedded with optimal cutting temperature (OCT) compound and coronal sections were prepared with cryostat (Leica) at 20 μm-thickness.

To perform immunofluorescence staining, brain sections and cell samples were washed with PBS, and then incubated with blocking buffer (PBS containing 3% goat serum and 0.1% Triton X-100) for 1 h at room temperature followed by incubation with primary antibodies at 4°C overnight. The second day, sections and cell sample slides were applied with DAPI (4′,6-diamidino-2-phenylindole) (D8417, Sigma) and fluorescence-labeled secondary antibodies for 1 h. For BrdU immunostaining, the sections and cell samples were pretreated with 1 M HCl in at 37°C for 30 min before blocking. The samples were viewed and images were taken with Olympus confocal microscope (Olympus IX83-FV3000). The antibody information was included in Table S3.

### Plasmids construction, transfection and lentivirus transduction

Mouse EphA2 cDNA, Scramble (5’-TTCTCCGAACGTGTCACGT-3’) and shRNAs targeting mouse *Zmynd11* (5’-TTGCCAACATTGATCGTATTA-3’) were cloned into pSicoR lentiviral vectors (pSicoR-TOMATO or pSicoR-GFP) for lentivirus packaging or pLKO.1 vector, respectively. Lentivirus was added to cultured eNPCs and neurons (DIV 4) (MOIs: 10), respectively. eNPCs were induced for differentiation 3 days post virus infection, and harvested for assays 48 h later. Neurons were harvested for assays at DIV 7.

For transfection of cultured neurons at DIV 14 and DIV 18, 2 μg plasmids, 2 μL Lipo2000 were mixed with 600 μL neurobasal medium. After incubated for 30 min, the mixture of 200 μL was added to each well in 24-well plate. The medium was replaced with fresh maintaining medium 4 h later and cells were harvested for assays 3 days later.

### Co-immunoprecipitation assay

Cells were lysed with lysis buffer (50 mM Tris–HCl pH 7.8, 150 mM KCl, 0.1% Triton X-100, 5 mM EDTA, 1 tablet of protease inhibitor/50 mL) for 30 min on ice followed by a centrifugation at 4°C. The supernatants were incubated with primary antibody at 4°C overnight. The second day, Protein A/G magnetic beads were added into samples and incubated for 6 h at 4°C. Ater washed with washing buffer (50 mM Tris–HCl pH 7.8, 150 mM KCl, 0.01% Triton X-100, 5 mM EDTA, 1 tablet of protease inhibitor/50 mL) for 10 min 3 times, the beads were resuspended with loading buffer. After incubated at 100°C for 5 min and cool down for 5 min on ice, the samples were applied for immunoblotting assay.

### In utero electroporation

In utero electroporation (IUE) was performed as described previously [44, 46]. C57/BL6 pregnant mice (E14.5) was anesthetized with isoflurane (RWD). Plasmids were mixed with Venus-GFP (ratio of 3:1) and 0.01% fast green, and manually microinjected into fetal lateral ventricle with a glass capillary (Hirchmann DE-M 16). Five 100-μs pulses of 35 V at 900-μs intervals were delivered across the uterus using an electroporator (BEX, SN. 101438). At E17, fetuses were dissected out and perfused with cold PBS followed by cold 4% PFA. Brain samples were removed out and fixed with 4% PFA overnight at 4°C. After dehydrated with 30% sucrose solution, brain samples were embedded with OCT, and sectioned coronally at a thickness of 15 μm. Brain sections were viewed and images were captured with confocal microscopy (OLYMPUS IX83-FV3000). Images were analyzed with ImageJ software.

### RNA extraction and qRT-PCR

Total RNA of brain tissues and cells were extracted with TRIzol reagent following manufacturer’s protocol (15596018, Invitrogen), respectively. The concentration of RNA was measured by NanoDrop spectrophotometer 2000 (Thermo Fisher Scientific) and 500 ng of total RNA was used to generate cDNA. qRT-PCR was made using SYBR Green qPCR mix (Q711, Vazyme) in triplicate, and measured by Applied Biosystems Viiia 7. The final results were calculated in 2^−△△ct^ method. The used primers for qRT-PCR were shown in Table S2.

### RNA sequencing

Samples used for the cDNA library were assessed by NanoDrop 2000 (Thermo Fisher Scientific). The RNA integrity value (RIN) was determined with the RNA Nano 6000 Assay Kit of the Bioanalyzer 2100 system (Agilent Technologies Inc.). A total amount of 3 μg RNA was used for each RNA sample preparations. Sequencing libraries were generated following manufacturer’s recommendations and index codes were added to attribute sequences to each sample using NEBNext UltraTM RNA Library Prep Kit for Illumina (NEB).

Raw reads of fastq format were processed to analyze RNA-seq data. Clean reads were gained from raw data by removing reads containing adapter and ploy-N. Then the remained clean reads were mapped to the *Mus musculus* genome (mm10) using Hisat2 v2.2.1. FeatureCounts v2.0.1 was used to count the read numbers mapped to each gene to quantify the gene expression level. Fragments per kilobase of transcript per million mapped reads (FPKM) of genes were calculated based on the length of the genes and the genes mapping of read counts. The analysis of differential expression was performed using the edgeR package (3.32.1). The p-values were adjusted with the Benjamini and Hochberg’s approach for controlling the false discovery rate. A p-value of 0.05 and absolute fold-change of 2 were set as the threshold for significantly differential expression.

### Histone extraction

Cortical tissues of new born pups (P0) were dissected and cut into small pieces. Tissues and cultured eNPCs were resuspended with 1 mL hypotonic lysis buffer (10 mM Tris–HCl, 1 mM KCl, 1.5 mM MgCl_2_, 1 mM DTT, 10 mM phosphatase inhibitor). The mixture was incubated at 4°C for 30 min and centrifuged for 10 min at a speed of 10000 g. The precipitation was resuspended with 200 μL 0.4 M H_2_SO_4_ was added and incubated at 4°C for 2 h. After centrifuged (16000 g for 30 min), the supernatants were collected, and 132 μL trichloroacetic acid was added followed by incubation at 4°C overnight. The second day, the mixture was centrifuged (16000 g, 30 min), and the precipitation was resuspended with 1 mL acetone followed by a centrifugation (16000 g, 4 min). The supernatants were discarded and 1 mL 95% ethanol was added followed by a centrifugation (16000 g, 5 min). The precipitation was dried at room temperature, and dissolved with nuclease free water followed by WB assays.

### H3K36me3 co-immunoprecipitation assay

eNPCs and cortical tissues were collected and lysed with 500 μL NP40 buffer 1 (150 mM NaCl, 1.5 mM MgCl_2_, 0.5% NP40, 50 mM Tris–HCl at pH8.0, 1 tablet protease inhibitor cocktail (P8340, Sigma) on ice for 30 min. Cell lysates were used for immunoprecipitation with H3K36me3 antibody (PTM-625, PTMab) and the corresponding IgG (B900610, Proteintech). Protein A/G magnetic beads (10002D, Thermo Fisher Scientific) were used for H3K36me3 IP. Protein A/G magnetic beads were washed 3 times with IP washing buffer (10 mM Tris–HCl pH7.5, 1 mM EDTA, 1 mM EGTA, 150 mM NaCl, 1% Triton-X, 0.2 mM sodium orthovanadate) and analyzed with western blotting.

### Chromatin immunoprecipitation-qPCR

ChIP experiment was performed following the manufacture’s protocol of the ChIP Kit (P2078, Biyuntian). The experimental procedure was conducted as follows: Freshly harvested cells were crosslinked with 1% formaldehyde in complete medium for 10 min at room temperature to stabilize protein-DNA interactions. The crosslinking reaction was quenched by adding 125 mM glycine for 5 min. Cells were subsequently washed three times with ice-cold phosphate-buffered saline (PBS, pH 7.4) and pelleted by centrifugation at 300 g for 5 min at 4 °C. Nuclear fractions were isolated using SDS-lysis buffer (50 mM Tris–HCl pH 8.1, 10 mM EDTA, 1% SDS) supplemented with protease inhibitor cocktail. Chromatin was fragmented to 200–500 bp fragments. Fragment size distribution was verified by 1.5% agarose gel electrophoresis. For immunoprecipitation, chromatin lysates were pre-cleared with Protein A/G agarose beads for 1 h at 4 °C. Subsequently, 2 μg of specific primary antibodies were added to chromatin lysate and incubated with rotation at 4 °C overnight. Antibody-chromatin complexes were captured by incubation with Protein A/G magnetic beads for 2 h at 4 °C. Immune complexes were sequentially washed. Bound chromatin was eluted using elution buffer (1% SDS, 100 mM NaHCO3) and reverse crosslinked at 65 °C overnight. Purified DNA was analyzed by quantitative PCR (qPCR) using SYBR Green Master Mix on a Real-Time PCR System. Primer sequences targeting specific genomic regions of interest were validated for amplification efficiency. Data were normalized to input controls and presented as percentage input using the ΔΔCt method. All experiments were performed in triplicate biological replicates.

### DNA pull-down

DNA template was labeled with Biotin-11-dUTP (86303-25-5, MedChemExpress) as manufacturer’s protocol (R001 A, Takara). DNA template labeled with biotin was verified by DNA gel electrophoresis. Desired DNA was extracted by VAHTS DNA clean beads (N411-01, Vazyme). Nucleic proteins were extracted from fresh cortical tissues with NE-PER Nuclear and cytoplasmic Extraction Reagents (78833, Thermo Scientific). 1 μg biotin-labeled DNA template was mixed with 70 μg nucleic protein samples and 50 μL Mag-SA beads (M1000S, BIOEAST). The mixture was incubated at 4°C for 1 h. The mixture was washed with ice-cold PBS for 3 times, and the beads were centrifuged (5000 g, 1 min). The beads were mixed with loading buffer and denatured at 100°C for 10 min followed by WB assays. The template probe primers could be found in Table S2.

### Western blot assay

Cells and brain tissues were homogenized in RIPA buffer (ab156034, Abcam) containing 1X protease inhibitor cocktail (P8340, Sigma) and centrifuged at 14,000 rpm for 30 min at 4 °C. The supernatants were collected and the concentration of protein was measured with Biophotometer (Eppendorf). 20–40 μg proteins of each sample was separated by sodium dodecyl sulfate–polyacrylamide gel electrophoresis (SDS-PAGE) according to the molecular size and then transferred onto a polyvinylidene fluoride membrane. The bands were blocked in 5% BSA (MB4219, Meilunbio) in TBST at 25 °C for 1 h and incubated with antibodies at 4 °C overnight. The signals of the targeted bands were incubated with UltraSignal ECL (4 AW001, 4 A Biotech), and detected by Tanon Detection system (Tanon 5200). The intensity was analyzed with Adobe Photoshop software.

### Statistical analysis

All data are expressed as mean ± SEM using GraphPad Prism 10 (GraphPad Software, CA). The number of independent biological replicates was described in figure legends. Quantitative RT-PCR, immunofluorescence staining and western blot assays were replicated at least three times. The differences were assessed using an unpaired Student’s t test between two groups; one-way and two-way ANOVA analysis followed by Tukey’s post hoc analysis were used to determine differences for comparison of multiple groups. Statistical significance was considered when p < 0.05 (statistical significance: n.s, no significance; *, p < 0.05; **, p < 0.01; ***, p < 0.001, ****, p < 0.0001).

## Supplementary Information

Supplementary material 1: Figure S1. Dynamic and abundant expression of Zmynd11 in embryonic brain and neuronal progenitor cells.Representative images of Zmynd11 and embryonic neuronal progenitor cellsmarker Nestin, neuronal cell marker Tuj1 immunostaining with brain sections of embryonic miceand postnatal day 0 mice. Scale bars, 50 μm. SVZ, subventricular zone; VZ, ventricular zone; IZ, intermediate zone. Representative images of WBand quantification resultsshowed that the level of Zmynd11 was significantly increased in the cortex with neuronal development of mice. GAPDH was used as an internal control. n = 5 mice in each group. Data are presented as mean ± SEM; *p < 0.05; **p < 0.01; ***p < 0.001, ****p < 0.0001, one-way ANOVA analysis followed by Tukey’s multiple-comparison test, F= 62.19. qRT-PCR assay results showed the level of *Zmynd11* was significantly increased in the cortex of mice with neuronal development. n = 3 biologically independent experiments. Data are presented as mean ± SEM; *p < 0.05, **p < 0.01, ***p < 0.001, one-way ANOVA analysis followed by Tukey’s multiple-comparison test, F= 116.3. Representative images of Zmynd11, Nestin and Tuj1 immunostaining with proliferating and differentiated eNPCs, respectively. Scale bars, 50 μm. Representative images of WB and quantification results showed that the level of Zmynd11 was significantly increased upon the differentiation of eNPCs. GAPDH was used as an internal control. n = 5 mice in each group. Data are presented as mean ± SEM; *p < 0.05; **p < 0.01; ***p < 0.001, ****p < 0.0001, unpaired Student’s t test.qRT-PCR assay results showed the level of *Zmynd11* was significantly increased upon the differentiation of eNPCs. n = 3 biologically independent experiments. Data are presented as mean ± SEM; *p < 0.05, **p < 0.01, ***p < 0.001, unpaired Student’s t-test.Representative images of Zmynd11, Nestin, neuronal cell marker MAP2 and astrocyte cell marker GFAP immunostaining with proliferating eNPCs, neurons and astrocytes respectively. Scale bars, 50 μm.Representative images of WBand quantification resultsshowed that the level of Zmynd11 in neurons and astrocytes was significantly increased compared to proliferating eNPCs. GAPDH was used as an internal control. n = 5 mice in each group. Data are presented as mean ± SEM; *p < 0.05; **p < 0.01; ***p < 0.001, ****p < 0.0001, one-way ANOVA analysis followed by Tukey’s multiple-comparison test, F= 61.14.qRT-PCR assay results showed the level of *Zmynd11* in neurons and astrocytes was significantly increased compared to proliferating eNPCs. n = 3 biologically independent experiments. Data are presented as mean ± SEM; *p < 0.05, **p < 0.01, ***p < 0.001, one-way ANOVA analysis followed by Tukey’s multiple-comparison test, F= 106.2. Figure S2. Specific deficiency of *Zmynd11 *in Nestin^+^ cells results in developmental deficits of mice. Schematic diagram of the generation of *Zmynd11* cKO mice.Representative images of Ctrl and cKO mice at the age of P0. Quantification of body weight of Ctrl and cKO mice at P0. n = 6 mice for each group. Data are presented as mean ± SEM; *p < 0.05, **p < 0.01, ***p < 0.001, unpaired Student’s t-test. Representative images of radial glial marker PAX6 immunostaining with scramble and *Zmynd11* KD brain sections. Scramble and sh*Zmynd11* plasmids were delivered into the brain via in utero electroporation at E14, and mice were sacrificed for assay at E17. Scale bar, 50 μm. Quantification results showed the percentage of PAX6^+^GFP^+^/GFP^+^ in VZ/SVZ layer was significantly decreased in *Zmynd11* KD group compared to Ctrl. n = 5 fetuses at least from 4 pregnant mice for each group. Data are presented as mean ± SEM; *p < 0.05; **p < 0.01, unpaired Student’s t test.Representative images of intermediate progenitor marker Tbr2 immunostaining with scramble and *Zmynd11* KD brain sections. Scale bar, 50 μm. Quantification results showed the percentage of Tbr2^+^GFP^+^/GFP^+^ in VZ/SVZ layer was significantly increased in *Zmynd11* KD group compared to Ctrl. n = 5 fetuses at least from 4 pregnant mice for each group. Data are presented as mean ± SEM; *p < 0.05; **p < 0.01, ***p < 0.001, unpaired Student’s t test. Representative images of the distribution of GFP^+^ cells in the cortex of E17 fetal brain. Scramble and sh*Zmynd11* plasmids were delivered into E14 mice brains *via* IUE, and mice were sacrificed for assays at E17. Scale bar, 50 μm. Quantification results showed that *Zmynd11* KD induced fewer GFP^+^ cells in VZ/SVZ and CP regions, but more cells in IZ compared to scramble group. n = 5 fetuses at least from 4 pregnant mice for each group. Data are presented as mean ± SEM; *p < 0.05; **p < 0.01, ***p < 0.001, unpaired Student’s t test. Figure S3. *Zmynd11* KD inhibits PI3K signaling pathway in eNPCs. Schematic diagram of genes relating to PI3K signaling pathway.qRT-PCR assay results showed that *Zmynd11* KD significantly reduced the mRNA level of multiple key components of PI3K signaling pathway in eNPCs. n = 3 biologically independent experiments. Data are presented as mean ± SEM; *p < 0.05, **p < 0.01, ***p < 0.001, n.s. no significance; unpaired Student’s t-test. Representative images of WB of PI3K components in proliferating eNPCs infected with lenti-scramble and lenti-sh*Zmynd11*, respectively. Cells were collected for assays 3 days post the virus infection. Quantification results showed that *Zmynd11* KD significantly reduced the level of Epha2. Although the levels of total IRS1, PI3K, PDPK1, and SGK1were not changed, the levels of Epha2, p-IRS1, p-PI3K, p-PDPK1, and p-SGK1were significantly decreased in eNPCs. n = 4 biologically independent experiments. Data are presented as mean ± SEM; *p < 0.05, **p < 0.01, ***p < 0.001, unpaired Student’s t-test. Figure S4. *Zmynd11* deficiency did not affect total levels of H3K36me3 and Pol II. Immunoprecipitation followed by WB assays showed that Zmynd11 easily precipitated H3K36me3 in eNPCs, and vice versa. ChIP followed by WB assays showed the enrichment of H3K36me3 at *Epha2* promotor in eNPCs.WB assays and quantification results showed that the level of total H3K36me3 was not changed in cKO brain tissues and *Zmynd11* KD eNPCs. H3 was used as internal control. n = 4 biologically independent experiments. Data are presented as mean ± SEM; *p < 0.05, **p < 0.01, ***p < 0.001, unpaired Student’s t-test.ChIP followed by WB assay showed the binding of RNA Polymerase II at *Epha2* promotor in eNPCs. WB assay and quantification results showed that levels of Pol II was not changed in cKO brain tissues. Tubulin was used as internal control. n = 4 biologically independent experiments. Data are presented as mean ± SEM; *p < 0.05, **p < 0.01, ***p < 0.001, unpaired Student’s t-test. Figure S5. Exogenous *Epha2* rescues neuronal deficits induced by *Zmynd11* KD. Cortical neurons from E14 fetal mice were infected with lentivirus at DIV 4, and MAP2 immunostaining was performed at DIV 7. Lenti-scramble-GFP, Lenti-RFP, Lenti-sh*Zmynd11*-GFP, and lenti-Epha2-RFP viruses were used for infecting cells. Scale bar, 50 μm. Quantification results showed that exogenous Epha2 significantly enhanced the dendritic lengthand the number of branch pointsof cortical neurons. n = 30 cells for each group. Data are presented as mean ± SEM; *p < 0.05, **p < 0.01, ***p < 0.001, one-way ANOVA analysis followed by Tukey’s multiple-comparison test, F= 44.25 for, F= 42.61 for. Sholl analysis results showed that exogenous Epha2 significantly increased the dendritic complexity of cortical neurons. n = 30 cells for each group. Data are presented as mean ± SEM; *p < 0.05, **p < 0.01, ***p < 0.001, one-way ANOVA analysis followed by Tukey’s multiple-comparison test, F= 10.53. qRT-PCR assay results showed that exogenous *Epha2* did not affect the level of *Zmynd11* mRNA, but significantly enhanced mRNA levels of *Epha2* and *Gfap*, and reduced the mRNA level of *Tuj1*. n = 3 biologically independent experiments. Data are presented as mean ± SEM; *p < 0.05, **p < 0.01, ***p < 0.001, one-way ANOVA analysis followed by Tukey’s multiple-comparison test, F= 82.44 for, F= 5374 for, F= 71.21 for, F= 107.8 for.

Supplementary material 2: Table S1. Differentially expressed genesin Zmynd11 KD eNPCs

Supplementary material 3: Table S2. The used primers

Supplementary material 4: Table S3. The used antibodies

## Data Availability

The plasmids and related data are available from the corresponding authors on reasonable request.
