# Supplementary figures and images for: Zmynd11 is essential for neurogenesis by coordinating H3K36me3 modification of Epha2 and PI3K signaling pathway
Source: Cell Biosci. 2025 Apr 25;15:55. doi: 10.1186/s13578-025-01392-z (PMC12032794; doi:10.1186/s13578-025-01392-z)

Figure S1

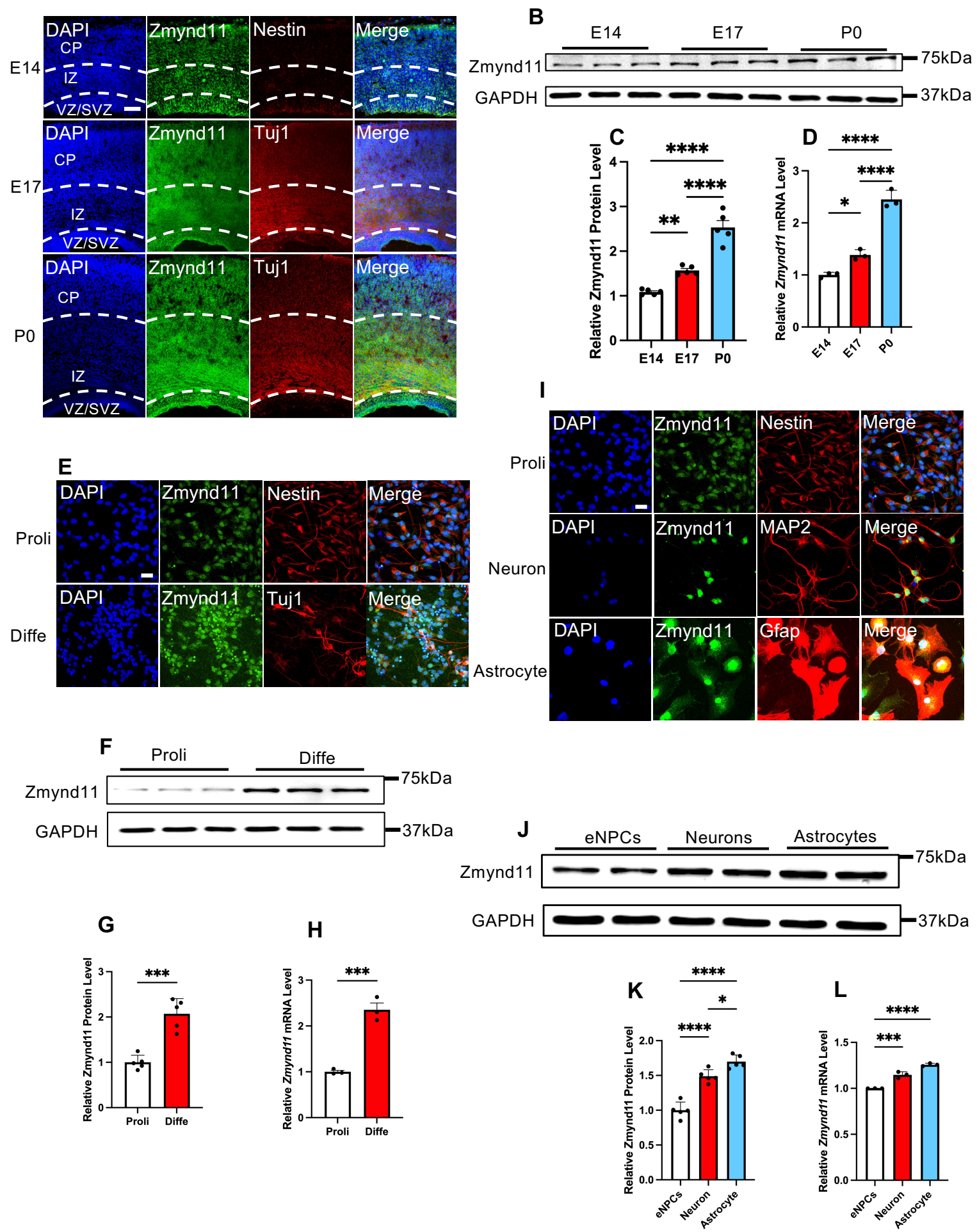

Figure S2

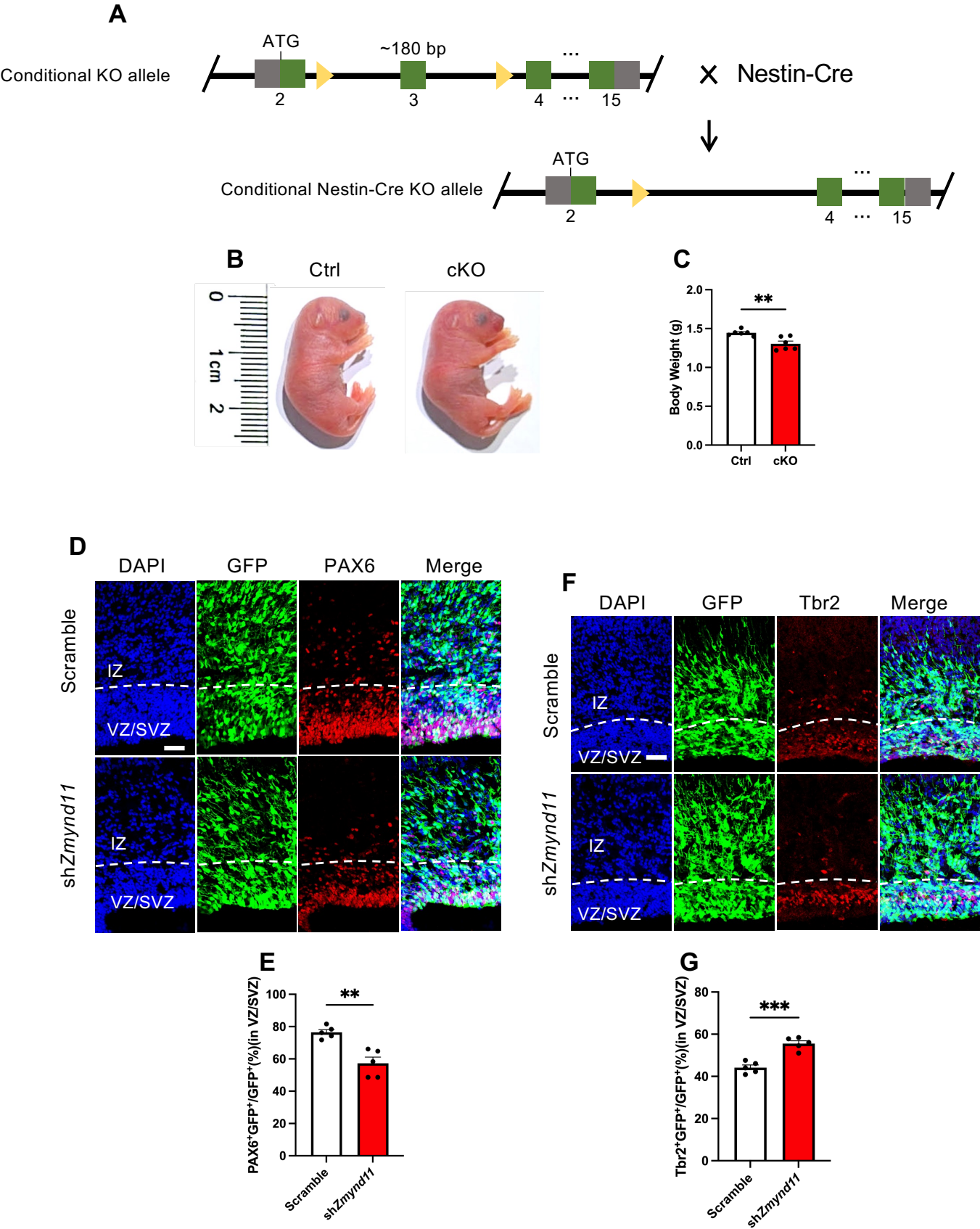

Continued Figure S2

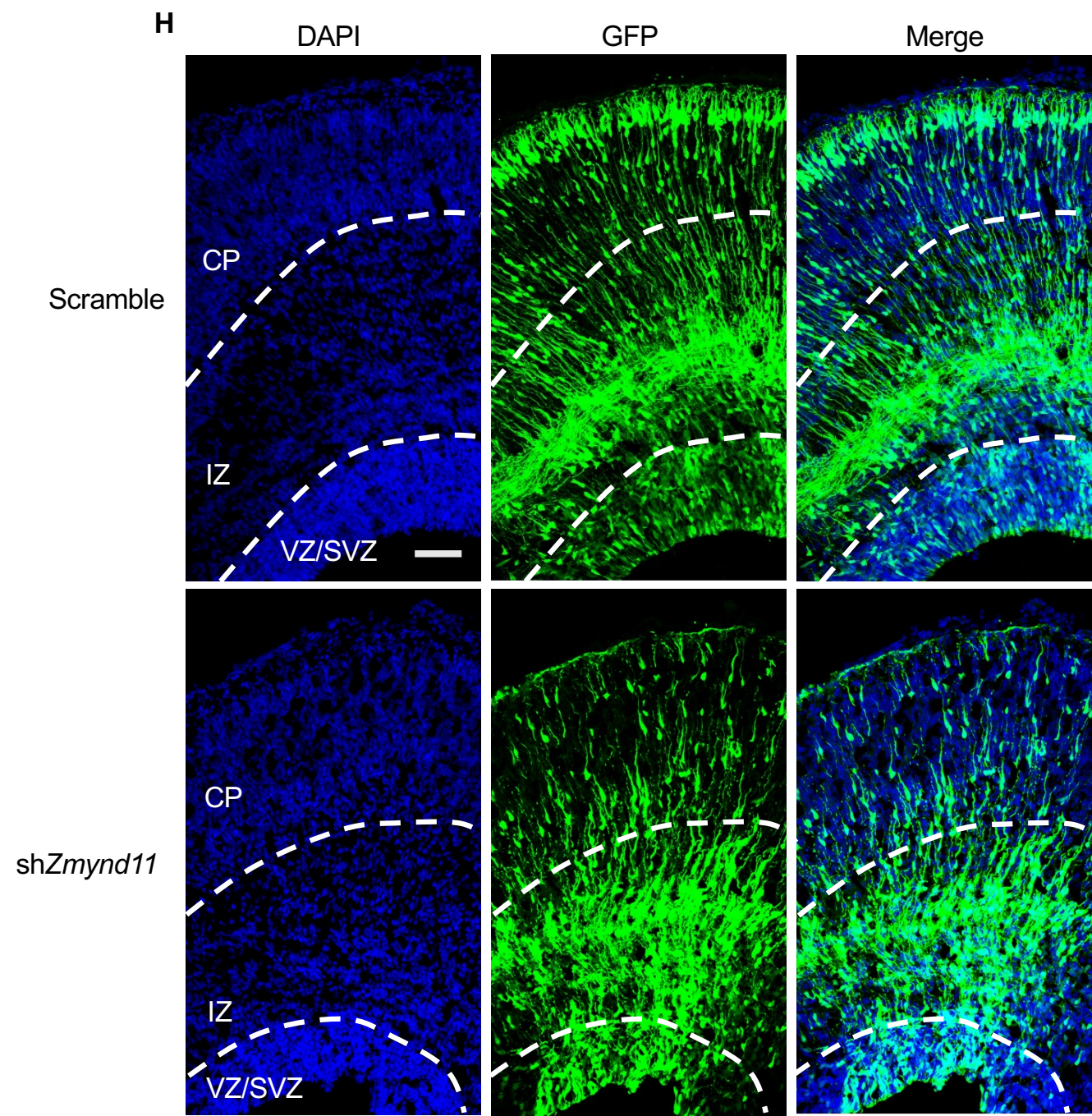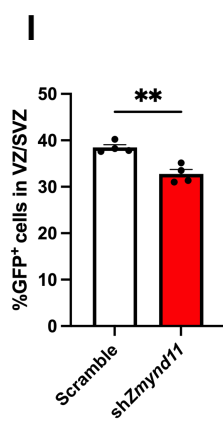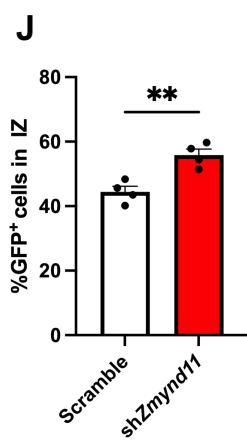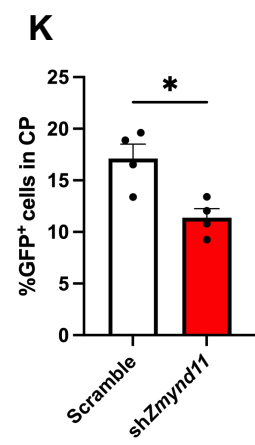

Figure S3

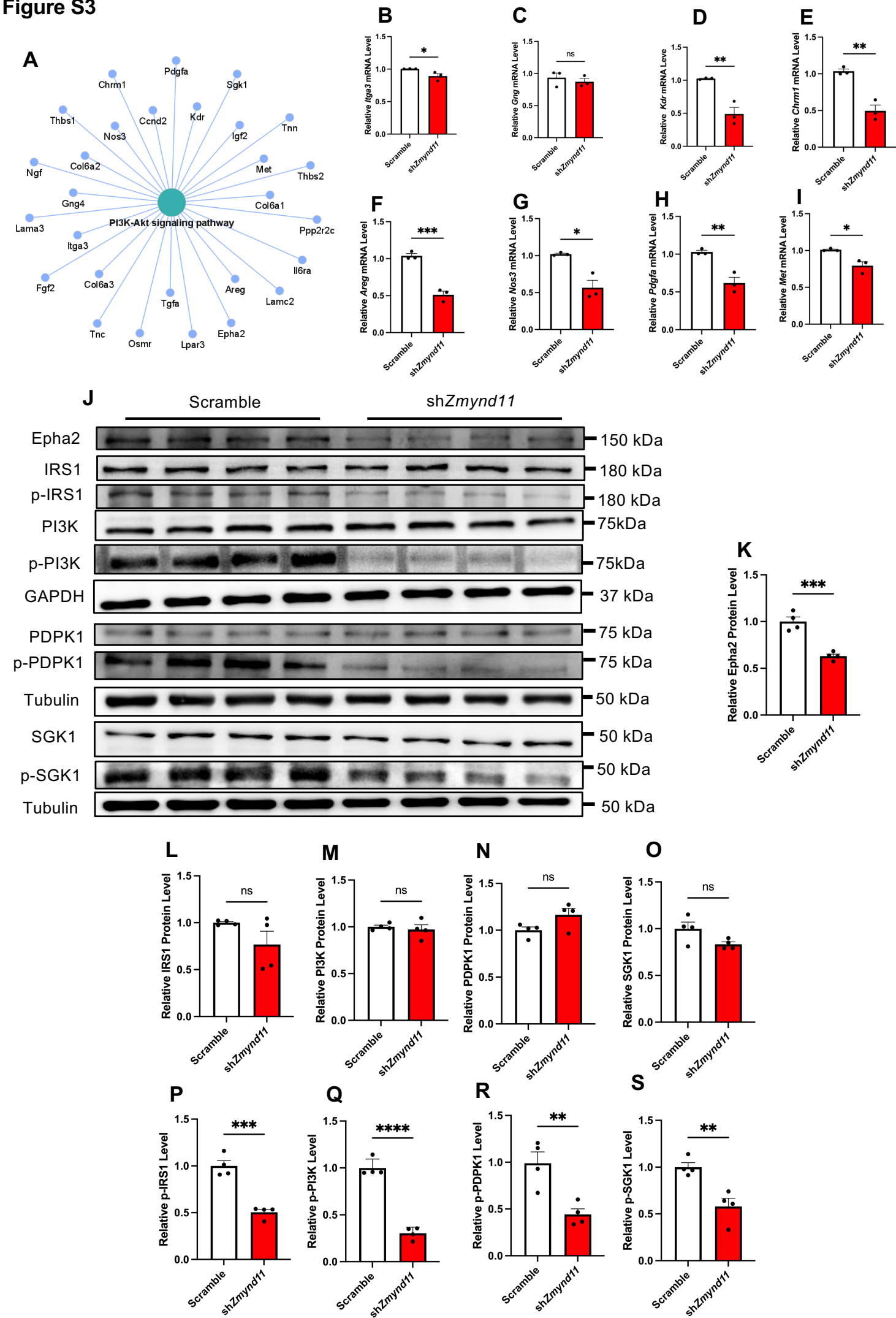

Figure S4

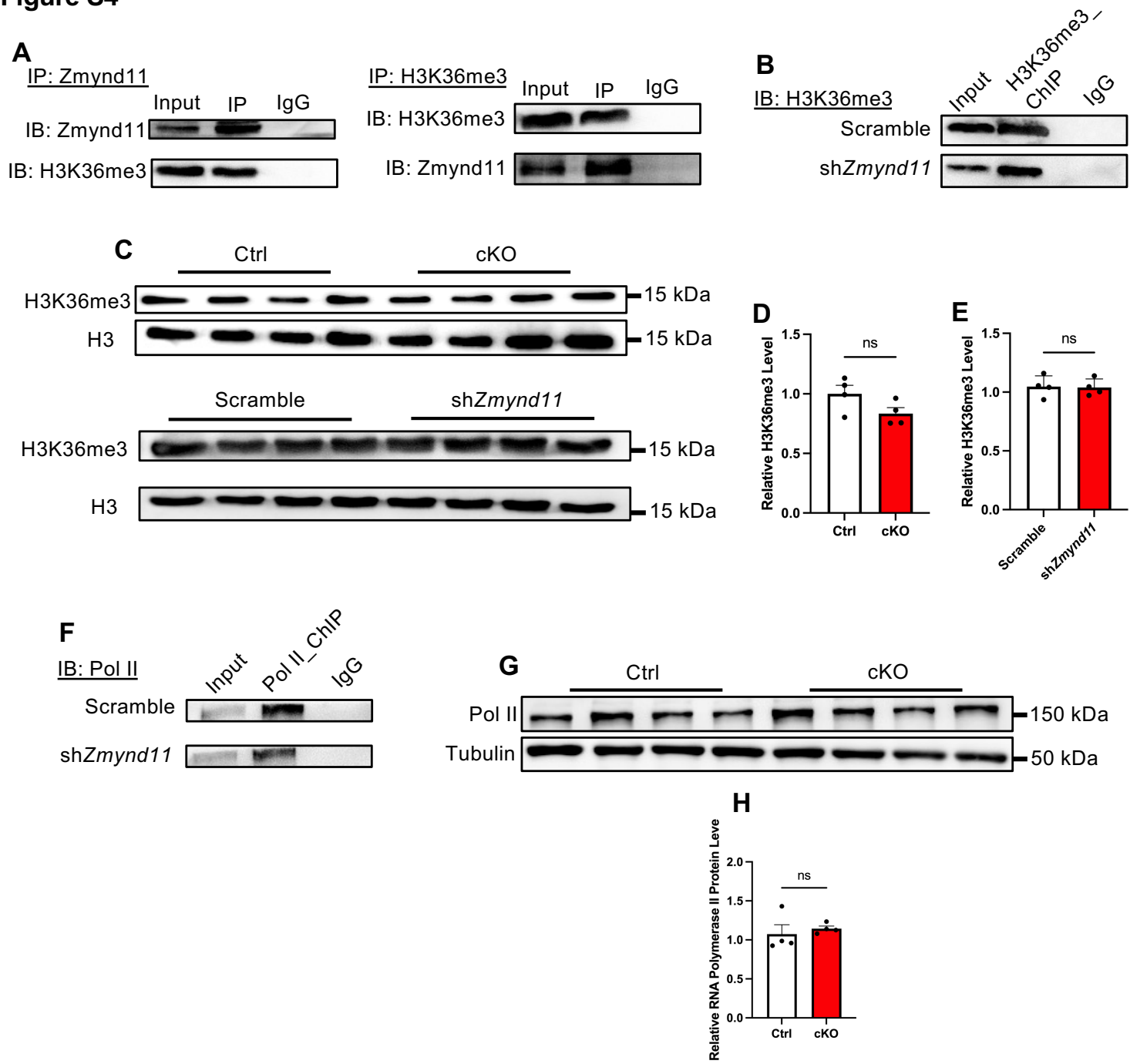

Figure S5

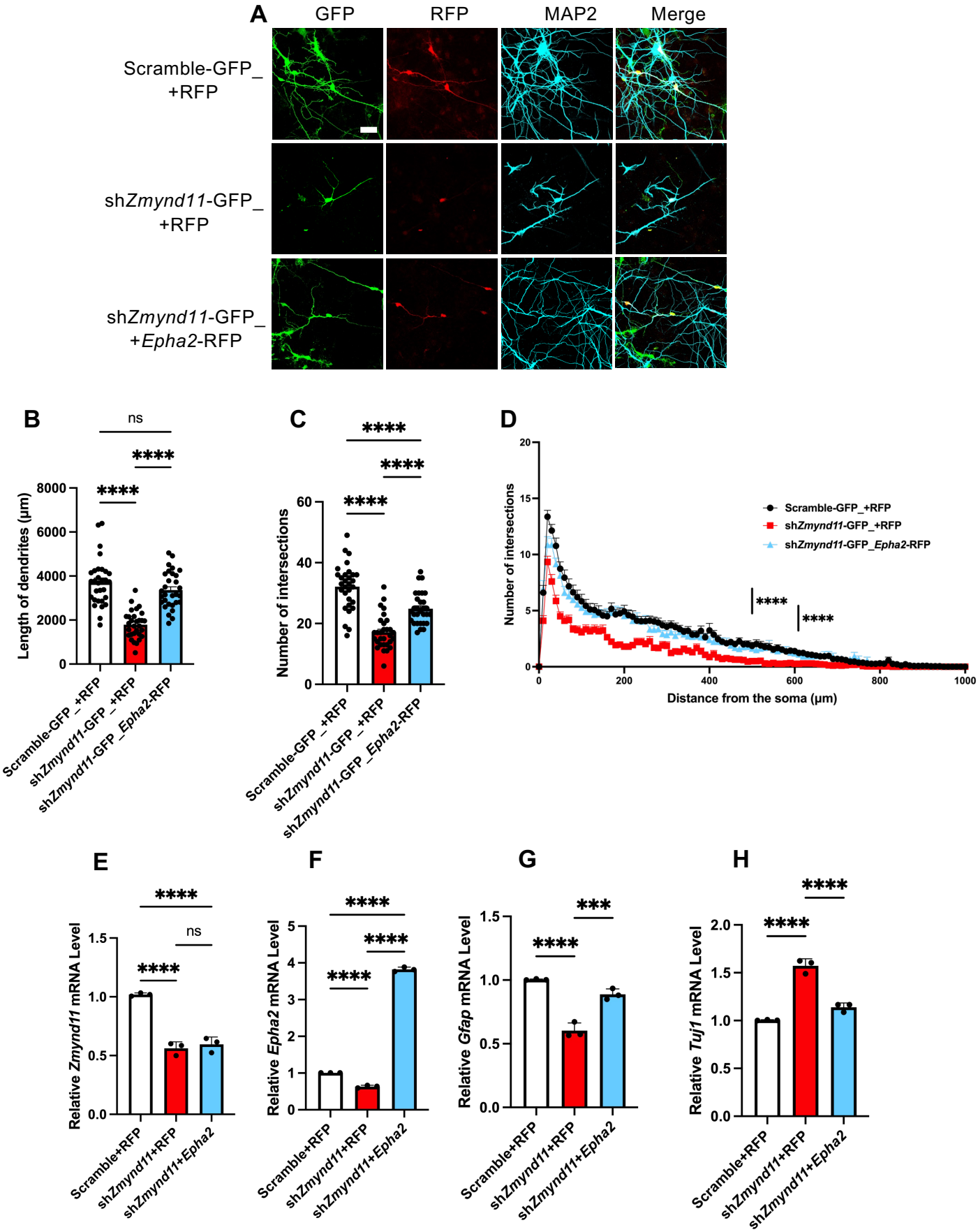

Supplement: Supplementary file 1 — Supplementary material 1: Figure S1. Dynamic and abundant expression of Zmynd11 in embryonic brain and neuronal progenitor cells.Representative images of Zmynd11 and embryonic neuronal progenitor cellsmarker Nestin, neuronal cell marker Tuj1 immunostaining with brain sections of embryonic miceand postnatal day 0 mice. Scale bars, 50 μm. SVZ, subventricular zone; VZ, ventricular zone; IZ, intermediate zone. Representative images of WBand quantification resultsshowed that the level of Zmynd11 was significantly increased in the cortex with neuronal development of mice. GAPDH was used as an internal control. n = 5 mice in each group. Data are presented as mean ± SEM; *p < 0.05; **p < 0.01; ***p < 0.001, ****p < 0.0001, one-way ANOVA analysis followed by Tukey’s multiple-comparison test, F= 62.19. qRT-PCR assay results showed the level of Zmynd11 was significantly increased in the cortex of mice with neuronal development. n = 3 biologically independent experiments. Data are presented as mean ± SEM; *p < 0.05, **p < 0.01, ***p < 0.001, one-way ANOVA analysis followed by Tukey’s multiple-comparison test, F= 116.3. Representative images of Zmynd11, Nestin and Tuj1 immunostaining with proliferating and differentiated eNPCs, respectively. Scale bars, 50 μm. Representative images of WB and quantification results showed that the level of Zmynd11 was significantly increased upon the differentiation of eNPCs. GAPDH was used as an internal control. n = 5 mice in each group. Data are presented as mean ± SEM; *p < 0.05; **p < 0.01; ***p < 0.001, ****p < 0.0001, unpaired Student’s t test.qRT-PCR assay results showed the level of Zmynd11 was significantly increased upon the differentiation of eNPCs. n = 3 biologically independent experiments. Data are presented as mean ± SEM; *p < 0.05, **p < 0.01, ***p < 0.001, unpaired Student’s t-test.Representative images of Zmynd11, Nestin, neuronal cell marker MAP2 and astrocyte cell marker GFAP immunostaining with proliferating eNPCs, neurons a [file 13578_2025_1392_MOESM1_ESM.pdf]
