# Supplementary material for: Zmynd11 is essential for neurogenesis by coordinating H3K36me3 modification of Epha2 and PI3K signaling pathway
Source: Cell Biosci. 2025 Apr 25;15:55. doi: 10.1186/s13578-025-01392-z (PMC12032794; doi:10.1186/s13578-025-01392-z)
Supplement: Supplementary file 2 — Supplementary material 2: Table S1. Differentially expressed genesin Zmynd11 KD eNPCs [file 13578_2025_1392_MOESM2_ESM.pdf]

Gene	P Value	log2FC	regulated
Sulf1	0.002442044	-1.051285981	down
Gm7846	0.011384122	1.023486423	up
Il1r1	4.80E-12	-1.564629673	down
Hecw2	1.77E-29	-1.237878689	down
Abca12	0.007266635	-1.505105711	down
Rufy4	1.07E-15	-3.087448481	down
Ptprn	1.39E-58	-2.145527445	down
Scg2	7.12E-92	-3.980176922	down
Ap1s3	0.000866307	-1.459328353	down
Nyap2	0.000201296	-1.604247693	down
Sp100	0.03100161	1.218511252	up
Ecel1	1.95E-27	-3.829297722	down
Ngef	8.81E-07	-1.444354892	down
Col6a3	1.68E-33	-4.914909013	down
Gm7967	0.000721094	1.625171689	up
Tmem37	0.000624853	-1.114683036	down
Gm35243	0.031103314	1.10985374	up
Cd55	0.012053768	-1.304803229	down
Ptprc	0.004129883	1.027557488	up
Rgs1	0.025429468	-1.740465682	down
Ptgs2	7.53E-05	-1.721058378	down
Nmnat2	4.05E-30	-1.032691736	down
Lamc2	0.00734717	-1.118215245	down
Rgs8	1.11E-27	-2.311674994	down
Rgs16	2.09E-18	-1.199027706	down
Tnn	9.90E-07	-2.078285428	down
Sell	0.009984616	2.021427726	up
Dusp27	0.00038022	2.077761965	up
Rgs4	1.07E-15	-1.546545302	down
Arhgap30	8.69E-05	1.754585051	up
Ifi203	0.019847681	-1.028331531	down
Kif26b	6.80E-09	-1.830765264	down
1700056E22Rik	0.019798445	1.195309314	up
Lpgat1	3.64E-45	-1.343560395	down
Sertad4	0.001845077	-1.23298959	down
G0s2	2.81E-09	1.142832573	up
A330023F24Rik	0.019357438	-1.174819625	down
Akap12	1.12E-30	-1.03403444	down
Grm1	1.73E-06	-1.030432921	down
Plagl1	3.12E-20	-1.166682187	down
LOC108167795	0.002696373	1.731061683	up
Cited2	2.18E-14	-1.027175203	down
Arfgef3	0.000101647	-1.629778599	down
Perp	1.43E-15	-1.432388762	down
Sgk1	4.22E-39	-1.495809856	down
Trdn	0.002395455	1.221102548	up
5930403N24Rik	0.002934971	1.184150926	up

Unc5b	2.16E-28	-1.13245457	down
Srgn	0.039879858	-1.104118483	down
Col6a2	4.21E-09	-1.872068811	down
Col6a1	1.71E-23	-2.03914056	down
Col18a1	1.51E-10	-1.401530843	down
Hmha1	0.024723871	1.278261829	up
Gamt	6.97E-28	1.186738456	up
Slc17a8	0.000460355	-1.781185372	down
Phlda1	5.40E-30	-1.337317259	down
Cpm	9.87E-05	-1.131112431	down
Irak3	0.000358568	-1.547166645	down
Hmga2	2.40E-117	-2.5377169	down
Osbp2	0.006687972	-1.219449616	down
Cobl	2.68E-14	-2.33265246	down
Dock2	0.001388682	1.496855211	up
Fbll1	0.010886478	-1.164756045	down
Nipal4	0.006045283	-1.362262351	down
Itk	0.008931349	1.196035124	up
Tgtp2	0.032167738	1.697633858	up
Ifi47	0.007205406	1.211328379	up
P4ha2	0.000752537	1.04606237	up
Sparc	1.07E-33	-1.105290487	down
Gria1	1.21E-21	-1.093141153	down
Wnt9a	0.008536029	-1.322141733	down
Map2k3os	0.003806808	-1.145532176	down
Kcnj12	0.000459839	-1.273679668	down
Myh2	0.001671546	-1.72356616	down
Myh4	0.000221107	1.303963202	up
Myh8	3.82E-17	1.260384702	up
Tmem107	9.13E-09	1.041670025	up
G630025P09Rik	0.009192433	1.130015551	up
2810408A11Rik	5.15E-05	1.216608471	up
Scarf1	0.006495472	-1.209580119	down
Pipox	3.89E-30	1.043150636	up
Sez6	2.03E-45	1.02405828	up
Unc45b	6.92E-05	2.11951396	up
Slfn1	0.001149473	2.108128548	up
Itga3	7.39E-09	-1.37791678	down
Nxph3	1.46E-07	-2.139567088	down
D030028A08Rik	1.20E-05	-1.370124102	down
Gpr179	1.06E-07	1.354418658	up
Ccr7	0.010463819	1.201034092	up
Ptges3l	2.07E-15	1.129771455	up
Adam11	4.43E-13	-1.539745691	down
Scn4a	0.047725316	1.089339979	up
Abca6	0.000668431	-1.277531183	down
Tk1	5.20E-33	1.212060866	up
Nptx1	8.49E-47	-1.575401043	down

Gm11770	0.007002363	1.119914681	up
0610009L18Rik	0.004730661	1.08517219	up
Dcxr	4.22E-16	-1.124426241	down
Hmga1-rs1	2.34E-12	-1.373069769	down
Slc16a3	5.42E-07	1.816446402	up
Kcnf1	8.29E-37	-1.754970904	down
Tpo	0.001760105	-1.847244498	down
Sstr1	0.002995898	-1.262553401	down
Gm40443	0.008892545	-1.036628397	down
Gm40462	0.002517247	-1.5553083	down
Actn1	9.72E-15	-1.018331353	down
Rgs6	0.000248298	-1.198464068	down
Syndig1l	0.014479873	1.06104586	up
Fos	1.99E-12	-1.025465381	down
Gm20604	9.05E-10	1.531874737	up
Dlk1	2.01E-16	-2.737707293	down
Meg3	0.000307774	-1.135881718	down
Rian	1.55E-06	-2.40870267	down
Ahnak2	1.69E-05	-1.618241021	down
Jag2	2.63E-12	-1.972610504	down
Akr1c14	0.000445922	-1.093237269	down
Zmynd11	5.50E-97	-1.31425988	down
Edaradd	0.002663499	-1.36089938	down
Gng4	0.002415695	-1.096840259	down
Gm11335	0.002368998	1.742598466	up
Irf4	1.05E-05	-2.620568253	down
Mylk4	0.001109536	1.605159548	up
Serpinb1a	4.25E-19	1.004704408	up
Gm4024	0.000147711	1.060394016	up
Gcnt2	4.00E-10	-1.608621714	down
Rnf182	2.78E-25	-1.057054459	down
Fgd3	4.69E-21	-1.33393767	down
S1pr3	1.77E-34	-1.690180739	down
Drd1	4.83E-07	-2.014636227	down
Fbxl21	9.91E-07	-1.580422607	down
Tgfbi	4.37E-22	-3.171914861	down
Kif27	0.005863401	-1.31860162	down
Adcy2	0.002137492	-1.037162992	down
Mir682	0.000685149	1.843836863	up
Hapln1	0.010136496	-1.068578322	down
Thbs4	0.022002106	1.063650826	up
Pde8b	0.006002762	-1.112684763	down
Gm41025	0.002594213	-1.018768385	down
Gm41037	0.000578986	1.778734655	up
Gm41055	0.010666793	-1.377367454	down
Fhit	0.000158873	-1.740331852	down
Anxa7	1.09E-17	-1.085118482	down
Myoz1	0.013650509	1.059319799	up

Prkcd	0.001829153	-1.156966391	down
Itih3	4.30E-06	-1.141961647	down
LOC108168146	0.002272249	-1.00715103	down
Lgals3	3.52E-05	-1.379957529	down
Slc35f4	0.015940871	1.12905613	up
Dhrs2	0.008674723	1.007907403	up
Rec8	0.003314316	-1.056943594	down
Tgm1	9.36E-07	-3.382334344	down
Gzmb	0.000293224	1.181871956	up
2410022M11Rik	0.004133067	1.08369977	up
Gjb6	1.43E-05	-1.371213144	down
Sox7	1.26E-07	-3.267287008	down
Ptk2b	6.33E-10	-1.1941547	down
Pnma2	3.36E-16	-2.379873962	down
Nefl	2.75E-18	-2.206244765	down
Nefm	6.32E-30	-2.400197274	down
Gm21685	0.004980447	-1.849464196	down
Gm30365	5.00E-06	-1.79700716	down
Lect1	0.021253556	-1.192111499	down
Klf5	0.000203596	-1.099405361	down
Scel	0.002239917	-1.700512189	down
Rap2a	1.62E-46	1.016590415	up
2610035F20Rik	0.004648852	1.142013835	up
Sepp1	1.61E-38	-1.156876349	down
Fyb	0.00906755	1.583822743	up
Osmr	2.67E-33	-2.247093293	down
Npr3	0.031293668	-1.115372126	down
Ctnnd2	2.21E-59	1.077559535	up
Baalc	5.35E-25	-3.913836627	down
Tmem74	0.00043482	-1.32733752	down
Ebag9	3.78E-18	-1.038603421	down
Samd12	8.70E-22	-1.224434731	down
Nov	9.45E-17	-3.110021573	down
Deptor	5.80E-16	-1.819601311	down
St3gal1	4.73E-20	-1.062462447	down
Fam135b	1.92E-35	-1.584194775	down
Ly6a	0.045535554	1.059019196	up
Ly6c2	1.20E-06	2.841073539	up
Pvalb	0.008284794	1.171529616	up
Il2rb	1.26E-05	1.742057105	up
Rac2	0.001576173	1.02314959	up
Cyth4	0.028014721	1.173784301	up
Cacna1i	2.48E-07	-2.110434117	down
Grap2	0.002853223	1.506128416	up
Mchr1	0.001187369	1.326932376	up
Sult4a1	4.31E-06	-1.114622675	down
Pnpla3	9.19E-13	1.169412259	up
LOC108168256	0.030676432	1.038007957	up

A130051J06Rik	0.005298735	1.116040084	up
Amigo2	7.19E-12	-1.795765826	down
Ddn	0.000415306	-1.101120759	down
Bin2	0.000265686	2.296459438	up
Galnt6	0.032086204	1.052089916	up
Tns2	1.65E-08	1.003946817	up
Itgb7	0.000322484	2.062296687	up
Socs1	0.002518189	-1.010716896	down
Gm9861	0.024435357	1.027735276	up
Il1rap	4.10E-44	-1.111005331	down
Gm41448	0.014205769	1.093305181	up
Gm41450	0.003575677	-1.447619899	down
Hcls1	0.010082991	1.049049845	up
Cd80	0.000394235	-1.10251058	down
Zdhhc23	0.000343285	-1.24520142	down
Btla	1.75E-16	-1.044654208	down
Filip1l	5.07E-05	-1.364047627	down
Cyyr1	7.74E-05	-1.22515604	down
Slc5a3	4.29E-67	-1.425129935	down
Fndc1	4.76E-22	-1.608251919	down
Gm41523	0.019143609	1.137511416	up
Thbs2	5.10E-29	-1.949150422	down
Gm3435	0.000103597	-1.045366297	down
Gm4786	0.003182184	1.282020624	up
Oaz1-ps	0.001884318	1.146126139	up
Zfp677	6.57E-07	-1.417011175	down
Arhgdig	0.000731091	-1.265639788	down
Hmga1	8.73E-20	-1.327628236	down
Scube3	1.74E-05	-1.860779803	down
Rasal3	0.014333069	1.133363659	up
Ltb	0.002751159	1.777899152	up
H2-Q7	0.000136193	1.013094494	up
Cdsn	4.63E-07	-2.409370818	down
F630040K05Rik	0.000219061	1.331480172	up
Dlk2	0.003631869	-1.213869058	down
Arhgap28	0.0038716	-1.505573338	down
Emilin2	3.26E-12	-1.252432909	down
Lbh	1.22E-132	-1.739423264	down
Tmem178	3.37E-12	1.112546741	up
Gm36279	0.001017004	1.111761214	up
Mkx	1.87E-05	-1.113459075	down
Lama3	4.58E-08	-1.454858277	down
Syt4	5.58E-21	-1.558393964	down
Tslp	2.41E-05	-1.299779912	down
Hbegf	1.30E-46	-2.137551107	down
Pcdhac1	0.007360035	-1.179493864	down
Gm32139	0.013799242	1.198789835	up
Smim3	0.000200273	-1.026494556	down

Synpo	3.04E-05	-1.123625084	down
Pdgfrb	0.004289481	1.59942467	up
Cidea	0.000306519	1.096441607	up
Rab27b	0.00058193	-1.120101106	down
Myo5b	1.23E-17	-2.248874736	down
Slc14a2	3.75E-19	-1.442705707	down
Mtl5	0.000339106	1.085599635	up
Tbc1d10c	0.025190596	1.034895763	up
Clcf1	6.15E-25	-3.06717514	down
Actn3	0.000227336	1.208489078	up
Fosl1	4.00E-33	-4.348761795	down
Rnaseh2c	5.00E-11	1.110883288	up
Gm42067	0.006010881	-1.129442493	down
Chrm1	0.001456675	-1.11250887	down
Cd5	0.007174659	-2.115594402	down
Cd6	0.015869448	-1.34089608	down
Ms4a4b	0.003351039	2.007820699	up
Ms4a6b	0.000354386	1.973763684	up
Gm31733	0.0411387	1.121268263	up
Prune2	1.52E-27	-1.287576291	down
Anxa1	1.37E-07	-1.366434533	down
Pip5k1b	4.49E-15	-1.255202715	down
Dock8	0.005845806	1.05835385	up
Glis3	8.94E-27	-1.463787538	down
Slc1a1	5.53E-08	-1.041208046	down
Il33	7.26E-25	-1.452063274	down
Acta2	5.96E-32	-2.807986333	down
Ifit1	5.10E-10	-1.251033502	down
Hhex	0.01163324	-1.490881394	down
Morf4l1b	5.00E-06	1.094362065	up
Gsto2	0.016755166	1.339700019	up
Dusp5	2.04E-12	-1.112848022	down
Nutf2-ps1	2.15E-09	1.060232299	up
Gm31734	0.028488451	1.02742416	up
Gfra1	2.45E-33	-1.711075092	down
LOC102632465	0.005127961	1.369661584	up
Gm37416	0.030389088	1.238813208	up
Gm17762	0.003270593	1.414286801	up
Otud1	3.62E-32	-2.574445326	down
Entpd2	0.010710385	-1.316940242	down
C8g	0.018380296	1.164187532	up
Agpat2	4.34E-05	-1.103068058	down
Gm32316	0.000648314	1.581421572	up
1700001O22Rik	0.001595456	1.375337364	up
Fam129b	6.08E-18	-1.208781179	down
Cutal	0.040393083	1.190348759	up
Neb	0.002288189	1.013588932	up
Acvr1c	0.000613169	-1.27402279	down

Pla2r1	0.000344068	-1.280470761	down
Gm34739	0.017781896	1.369308347	up
Klhl41	0.006645712	1.21554438	up
Pdk1	3.20E-11	1.072558056	up
Neurod1	0.002086266	-1.362553815	down
Ppp1r1c	0.003703077	1.188670237	up
Pde1a	1.50E-21	-2.068291545	down
Gm19426	0.001189339	1.217385817	up
Serping1	0.001851667	-1.742530413	down
Mdk	0.005902512	1.124504248	up
Chst1	0.00129207	-1.627719363	down
Syt13	3.40E-21	-3.411692718	down
Tspan18	2.51E-07	-1.549675115	down
Cd44	4.06E-29	-1.181756113	down
Prrg4	2.08E-10	-1.283646235	down
Muc15	2.15E-07	-1.223928491	down
Actc1	8.75E-07	-1.189162501	down
Rasgrp1	0.006626097	1.115017632	up
Thbs1	1.24E-12	-1.341035285	down
Chac1	1.10E-05	1.04641771	up
Dusp2	0.003004417	-1.326107823	down
Gm14005	9.22E-07	-2.64232164	down
Slc4a11	5.63E-06	-1.477033776	down
Adra1d	2.42E-10	-1.863105192	down
Bmp2	2.42E-05	-1.316223774	down
Tmx4	6.06E-44	-1.094372385	down
Pak7	0.012657915	-1.097475373	down
Ism1	0.000699065	-1.202894366	down
Nkx2-2os	0.000109031	1.04252273	up
6430503K07Rik	0.009828851	1.167495278	up
Syndig1	0.006565823	-1.118096259	down
Mylk2	0.000449746	1.459099026	up
Necab3	0.003350124	1.21003675	up
Epb41l1	1.33E-28	-1.175840765	down
Cyp24a1	0.000188736	-1.701672926	down
Zbp1	0.000436353	1.845342159	up
Gm11007	0.003004832	2.152095921	up
Gm6710	1.01E-05	-1.644306724	down
Kcnq2	0.039340714	-1.040466665	down
Stmn2	0.001222094	1.351723176	up
Gm42187	0.041837071	1.115873678	up
Mccc1os	0.002802612	-1.421377681	down
Gm12532	1.21E-05	1.618213152	up
Fgf2	0.000106411	-1.164018204	down
Maml3	9.99E-06	-1.304943519	down
LOC108168870	0.000360584	-2.539615711	down
Gm32847	0.000234036	-1.776090469	down
Vmn2r1	0.002347362	-1.006823348	down

Tmem79	0.001314105	1.161540813	up
Il6ra	7.26E-19	-1.879584351	down
Rps27	2.70E-07	1.069534155	up
Tchh	0.001033315	-1.048623654	down
Tuft1	0.000148175	-1.240142397	down
Adamtsl4	0.004114324	-1.207123871	down
Cd2	0.000487435	1.536260032	up
Ngf	4.33E-12	-2.890539356	down
Syt6	1.64E-17	-2.161280154	down
Prok1	1.12E-09	1.398685113	up
Kcnc4	2.30E-12	1.144651131	up
Slc6a17	6.35E-53	-1.825260479	down
A730020M07Rik	0.031348279	-1.03243304	down
Abca4	0.000116726	-1.442059548	down
Ccdc109b	0.00222074	-1.149327798	down
Gbp5	0.015688973	1.100399558	up
Cyr61	1.29E-11	-1.161605606	down
Lpar3	0.000158905	-2.408652475	down
Ptgfr	3.10E-05	-1.117992721	down
Ak5	2.35E-16	-1.1942685	down
Penk	6.78E-11	-4.067906849	down
Srsf12	1.95E-11	-1.261296313	down
Gm26881	0.006000348	1.293846811	up
Tbc1d2	2.64E-05	-1.086254564	down
Gabbr2	1.16E-07	-1.360567936	down
Tmeff1	1.25E-12	-1.007157112	down
Ppp3r2	1.15E-05	1.084749953	up
Tnc	1.56E-33	-1.020643014	down
Podn	6.49E-06	-1.076355308	down
LOC105244657	0.000360183	1.254476982	up
Hivep3	4.32E-20	-1.049646513	down
Cited4	0.000275549	1.344056975	up
Hpcal4	6.46E-09	-2.701122727	down
Nt5c1a	0.005627494	-1.603195872	down
Sync	0.001015606	-1.027682658	down
Gm12992	2.75E-06	-1.083386118	down
Grhl3	0.000369729	-1.549978758	down
E2f2	2.07E-26	1.198286662	up
Kif17	1.93E-06	-1.363603308	down
Epha2	1.58E-32	-2.122761621	down
Efhd2	5.07E-22	-1.456703905	down
Tnfrsf1b	0.016802696	1.337119713	up
Gm13248	1.77E-05	-2.017829582	down
Tnfrsf9	0.011965755	1.328868097	up
Espn	4.38E-05	-1.040568888	down
Hes5	5.25E-13	1.050743207	up
Gm10560	1.20E-05	1.067093209	up
Cldn12	2.60E-28	-1.045938503	down

Gm8773	1.64E-23	-6.39069967	down
Cfap69	1.35E-05	-1.142455437	down
Steap2	1.60E-19	-1.125058286	down
Steap1	4.55E-11	-2.336855881	down
Sema3e	4.89E-62	-4.242089235	down
Nos3	4.64E-09	-2.877853529	down
Gm7420	0.005098305	1.169739714	up
Kcnk3	8.80E-16	-1.882737978	down
Spon2	0.013614857	-1.343926062	down
Ppp2r2c	1.36E-22	-1.355163701	down
Shisa3	1.34E-16	-1.101581319	down
Kdr	5.26E-06	-1.311443197	down
Gm42162	0.019503639	1.169295975	up
Areg	1.07E-06	-1.184050068	down
Cdkl2	0.001248224	-1.250926844	down
Prkg2	6.17E-15	-1.184498224	down
Gbp4	0.025378717	1.067342878	up
Gm17202	0.003462416	-1.438609599	down
Gm10419	0.000642967	-1.277696127	down
Slc26a1	0.002242268	-1.06577227	down
Myo18b	0.004085076	1.170412838	up
Selplg	0.000485094	1.822582059	up
Oasl2	8.25E-05	-1.221005804	down
Oasl1	0.000233139	-1.357051178	down
Srrm4	0.008615781	-1.226693138	down
Glt1d1	0.005009611	-1.14143882	down
Vgf	2.55E-100	-4.913476121	down
Serpine1	2.92E-07	-2.688905055	down
Pdgfa	1.14E-08	-1.063462414	down
Prkar1b	4.98E-08	-1.229433564	down
Col28a1	0.000714817	-1.181977958	down
Tes	0.001488808	-1.14456556	down
LOC108167381	1.04E-11	-1.929222649	down
Cav1	3.55E-44	-1.811899377	down
Met	6.43E-39	-2.572086799	down
Wnt2	8.11E-14	-2.914967904	down
Flnc	1.61E-10	-1.035510693	down
Kcp	0.008823408	-1.204057261	down
Podxl	1.09E-16	-1.323235364	down
Akr1b8	4.92E-08	-2.202582653	down
Gimap4	0.00154217	1.520961857	up
Gimap6	0.00860265	1.133262098	up
Gimap3	0.000399814	1.394861282	up
Gpnmb	3.34E-10	-1.175619576	down
Npy	0.002104183	-1.085286889	down
Il12rb2	0.008675839	1.927054125	up
Cd8a	0.00067406	1.948238536	up
Eva1a	0.000166913	-1.992659294	down

Dysf	9.76E-13	-1.419834689	down
Nat8	0.012505379	1.098828022	up
Tgfa	5.48E-24	-1.141099435	down
Pparg	0.003131137	-1.242234802	down
Alox5	1.13E-08	-1.223845417	down
Iqsec3	0.004217074	-1.295079076	down
Gapdh	6.35E-33	1.693799978	up
Kcna1	0.003245796	-1.257961765	down
Ccnd2	2.61E-106	-1.20397225	down
Tead4	1.74E-23	-4.087964185	down
Emp1	3.77E-23	-1.621082889	down
Arhgdib	0.000671066	1.29350357	up
Rerg	1.41E-17	-1.788034668	down
Pde3a	8.06E-37	-1.287603551	down
Rep15	0.005606204	-1.362098244	down
Cacng6	0.002216969	1.52798677	up
Il11	1.09E-10	-3.763885447	down
LOC105242405	0.000789648	-1.103451882	down
Hif3a	7.14E-08	-1.04938547	down
Gpr4	0.008465849	-1.14650481	down
Fosb	1.03E-11	-1.714483048	down
Ckm	0.000760716	1.138495437	up
Plaur	1.37E-15	-2.60959244	down
Pou2f2	0.009526538	1.033398622	up
Fbxo27	0.020754803	-1.156933361	down
Ryr1	0.000199207	1.14160241	up
Syt3	0.00187516	-1.023583366	down
Mybpc2	0.00044808	1.282926766	up
Slc17a7	0.000596553	-1.088544309	down
Ccdc155	0.001105734	1.117724848	up
Emp3	0.001303745	-1.052674819	down
Ipw	0.008295864	1.257010113	up
Lrrk1	1.42E-21	-1.260757857	down
Aldh1a3	1.67E-110	-5.033544801	down
Sv2b	3.01E-09	-2.474537324	down
Acan	2.73E-06	-1.931769686	down
Hapln3	0.035072811	-1.092085021	down
Pde8a	1.33E-06	-1.046526548	down
Cpeb1	9.16E-12	-1.235298217	down
Gm40448	0.003224166	1.187954972	up
Tenm4	5.28E-14	-1.126462206	down
P2ry2	1.44E-06	-2.15683902	down
Pde2a	1.49E-101	-2.616032044	down
Art1	0.031250262	1.071432932	up
Trim12c	0.000147263	-1.082125331	down
Gm8995	0.000312053	1.661205708	up
Cyb5r2	0.000193915	1.212604069	up
Rras2	6.85E-09	-1.014662122	down

Pdzd9	0.008110017	1.117582301	up
Hs3st2	0.003762427	1.037018003	up
Atp2a1	0.00065981	1.13394175	up
Spn	0.000245058	1.38042212	up
Kndc1	0.000176182	-1.384959995	down
Tnni2	0.011012199	1.002585672	up
Igf2	1.77E-07	-1.293584799	down
Gm33532	0.000220848	-2.137421597	down
Dlc1	3.39E-27	-1.239136619	down
Pdlim3	4.88E-05	1.457242376	up
Ankrd37	7.74E-06	1.421386563	up
Neil3	7.83E-12	1.002252255	up
Npy1r	1.01E-17	-1.662017244	down
Palm3	0.000477457	-1.604756015	down
Il27ra	0.046280923	1.046325762	up
Junb	9.10E-23	-1.038478049	down
Adcy7	0.037876169	1.022971325	up
Ccl22	0.002509248	-1.280454037	down
Plcg2	6.96E-06	-1.037695687	down
Piezo1	3.49E-29	-1.04214045	down
Acta1	0.003415391	1.055115216	up
Fut4	0.000219548	-1.096795626	down
Icam1	9.19E-05	-1.23524206	down
Fdx1l	0.047679049	1.058329175	up
Cnn1	1.56E-08	-3.717916559	down
Kirrel3os	0.000924254	-1.629898237	down
Nrgn	1.09E-18	1.29034006	up
Thy1	8.36E-10	1.089786524	up
Amica1	0.035626312	1.333953716	up
Tagln	1.61E-11	-2.36135299	down
Ttc12	8.12E-06	-1.240305451	down
Chrna5	0.007817169	-1.371964577	down
Mapk6	1.45E-55	-1.07884547	down
Snap91	1.27E-20	-1.116701413	down
Rasgrf1	9.09E-17	-2.912410374	down
Clstn2	2.21E-22	-1.01090888	down
Kif9	0.010178682	-1.012163202	down
Tmie	0.000111811	1.300742218	up
Trank1	0.016401862	-1.105363688	down
Stac	0.002756651	-1.408036645	down
Susd5	1.06E-05	-1.148865326	down
Scn5a	0.008867885	-1.018677802	down
Scn11a	0.00223791	-1.058425534	down
Mobp	0.020482628	-1.127338116	down
Clec3b	0.008585961	1.20856528	up
Tmem158	1.35E-29	1.340946112	up
ATP6	0.00043681	-1.351088486	down
ND4L	0.001193077	-1.224696804	down

Timp1	1.92E-08	-1.379727089	down
B230118I11Rik	0.017949184	-1.010165263	down
Cited1	2.72E-13	-1.348008479	down
Gm39529	0.000896325	1.315488179	up
P2ry10	0.006881949	1.227379389	up
Irs4	2.26E-34	-4.781094246	down
Rgag1	0.038326406	-1.009022594	down
Ptchd1	3.27E-07	-1.355488612	down
G530011O06Rik	0.007211859	-1.463404382	down
