# Supplementary material for: Zmynd11 is essential for neurogenesis by coordinating H3K36me3 modification of Epha2 and PI3K signaling pathway
Source: Cell Biosci. 2025 Apr 25;15:55. doi: 10.1186/s13578-025-01392-z (PMC12032794; doi:10.1186/s13578-025-01392-z)
Supplement: Supplementary file 3 — Supplementary material 3: Table S2. The used primers [file 13578_2025_1392_MOESM3_ESM.docx]

| **Primers for ChIP** | **sequence** |
| --- | --- |
| Fw-Epha2 promoter-1 | TGACATGTTCCCAGAGGCAC |
| Rv-Epha2 promoter-1 | TTTAAAGGGGCCGGACTGAC |
| Fw-Epha2 promoter-2 | CTTCACAGCGGGCTTTTTCC |
| Rv-Epha2 promoter-2 | GCACACACCCTCATGGGTAA |
| Fw-Epha2 promoter-3 | TCTTGAAGCCCAAGGGCAAA |
| Rv-Epha2 promoter-3 | TCGTGTGCAAACCTTGAGG |
| Fw-Epha2 promoter-4 | CCCAGCCTAGCGTATCCAAC |
| Rv-Epha2 promoter-4 | AGCCCGCTGTGAAGATCAAA |
| Fw-Epha2 promoter-5 | CCAGCCTAGCGTATCCAACA |
| Rv-Epha2 promoter-5 | AAGCCCGCTGTGAAGATCAA |
| Fw-Epha2 promoter-6 | TCCTCAAGGTTTGCACACGA |
| Rv-Epha2 promoter-6 | CTTTAAAGGGGCCGGACTGA |
| Fw-Epha2 promoter-7 | TGGTTAAAGCAGGTCAGGGC |
| Rv-Epha2 promoter-7 | GTCTTTAAAGGGGCCGGACT |
| **Primers for qRT-PCR** | **sequence** |
| Fw-Ccnd2-ms | GAGTGGGAACTGGTAGTGTTG |
| Rv-Ccnd2-ms | CGCACAGAGCGATGAAGGT |
| Fw-Col6a1-Ms | CTGCTGCTACAAGCCTGCT |
| Rv-Col6a1-Ms | CCCCATAAGGTTTCAGCCTCA |
| Fw-Itga3-Ms | CGATGGCTTCTCAGACGTG |
| Rv-Itga3-Ms | CAGCCCGCTTGTTGATGTTG |
| Fw-Gng4-Ms | GGCATGTCTAATAACAGCACCA |
| Rv-Gng4-Ms | CACTGGGATGATGAGGGGG |
| Fw-Osmr-Ms | CATCCCGAAGCGAAGTCTTGG |
| Rv-Osmr-Ms | GGCTGGGACAGTCCATTCTAAA |
| Fw-Nos3-Ms | GGCTGGGTTTAGGGCTGTG |
| Rv-Nos3-Ms | CTGAGGGTGTCGTAGGTGATG |
| Fw-Lama3-Ms | ACACCTGGGACGTGGATTG |
| Rv-Lama3-Ms | CTTGCAGGGTGAATGCTTCAT |
| Fw-Ngf-Ms | CCAGTGAAATTAGGCTCCCTG |
| Rv-Ngf-Ms | CCTTGGCAAAACCTTTATTGGG |
| M-Tuj1-Fw | TAGACCCCAGCGGCAACTAT |
| M-Tuj1-Rv | GTTCCAGGTTCCAAGTCCACC |
| Fw-epha2-ms | GCACAGGGAAAGGAAGTTGTT |
| Rv-epha2-ms | CATGTAGATAGGCATGTCGTCC |
| Fw-Efna2-Ms | CGATACGCAGTCTACTGGAAC |
| Rv-Efna2-Ms | GGTAGTCGTTGATGCTCACCT |
| Fw-Irs1-Ms | CGATGGCTTCTCAGACGTG |
| Rv-Irs1-Ms | CAGCCCGCTTGTTGATGTTG |
| Fw-Pi3k3r-Ms | TACAATACGGTGTGGAGTATGGA |
| Rv-pik3r3-Ms | GAGTCATTGGCTTAGGTGGCT |
| Fw-Pdpk1-Ms | GTGCCCATTCAGTCCAGTGT |
| Rv-Pdpk1-Ms | AAGGGGTTGGTGCTTGGTC |
| Fw-Sgk1-Ms | CTGCTCGAAGCACCCTTACC |
| Rv-Sgk1-Ms | TCCTGAGGATGGGACATTTTCA |
| M-ZMYND11-Fw | ATGAGGGGAAGTACCGGAGT |
| M-ZMYND11-Rv | CCTGCTCACTGTCTGCTCC |
| M-GAPDH-Fw | AGGTCGGTGTGAACGGATTTG |
| M-GAPDH-Rv | TGTAGACCATGTAGTTGAGGTCA |
| M-Nestin-Fw | CCCTGAAGTCGAGGAGCTG |
| M-Nestin-Rv | CTGCTGCACCTCTAAGCGA |
| M-GFAP-Fw | CCCTGGCTCGTGTGGATTT |
| M-GFAP-Rv | GACCGATACCACTCCTCTGTC |
| M-Cdk2-Fw | CCTGCTTATCAATGCAGAGGG |
| M-Cdk2-Rv | TGCGGGTCACCATTTCAGC |
| M-Cdk4-Fw | ATGGCTGCCACTCGATATGAA |
| M-Cdk4-Rv | TCCTCCATTAGGAACTCTCACAC |
| Fw-Cdk1-Ms | AGAAGGTACTTACGGTGTGGT |
| Rv-Cdk1-Ms | GAGAGATTTCCCGAATTGCAGT |
| Fw-s100b-Ms | TGGTTGCCCTCATTGATGTCT |
| Rv-s100b-Ms | CCCATCCCCATCTTCGTCC |
| Fw-cdk8-Ms | CGGGTCGAGGACCTGTTTG |
| Rv-cdk8-Ms | TGCCGACATAGAAATTCCAGTTC |
| Fw-chrm1-ms | AGTCCCAACATCACCGTCTTG |
| Rv-chrm1-ms | CAGGTTGCCTGTCACTGTAGC |
| Fw-Pdgfa-Ms | GAGGAAGCCGAGATACCCC |
| Rv-Pdgfa-Ms | TGCTGTGGATCTGACTTCGAG |
| Fw-Areg-Ms | GGTCTTAGGCTCAGGCCATTA |
| Rv-Areg-Ms | CGCTTATGGTGGAAACCTCTC |
| Fw-Met-Ms | GTGAACATGAAGTATCAGCTCCC |
| Rv-Met-Ms | TGTAGTTTGTGGCTCCGAGAT |
| Fw-Nos3-Ms | GGCTGGGTTTAGGGCTGTG |
| Rv-Nos3-Ms | CTGAGGGTGTCGTAGGTGATG |
| Fw-Kdr-Ms | TTTGGCAAATACAACCCTTCAGA |
| Rv-Kdr-Ms | GCAGAAGATACTGTCACCACC |
| **Primers for genotyping** | **sequence** |
| T052261-F1 | AAAGCATGGAGACATGACAAGTCTG |
| T052261-R1 | AAGTGACAGAGAAGTACCCAATGCC |
| T052261-F2 | CTATTTCAGGGCAGGGCAGTGTTT |
| T052261-R2 | CAGGAGCAGTCACAGCTACAGCTAG |
| **Primers for probe** | **sequence** |
| Fw-Epha2 -probe | TGGTTAAAGCAGGTCAGGGC |
| Rv-Epha2-probe | GTCTTTAAAGGGGCCGGACT |
