# Supplementary material for: Zmynd11 is essential for neurogenesis by coordinating H3K36me3 modification of Epha2 and PI3K signaling pathway
Source: Cell Biosci. 2025 Apr 25;15:55. doi: 10.1186/s13578-025-01392-z (PMC12032794; doi:10.1186/s13578-025-01392-z)
Supplement: Supplementary file 4 — Supplementary material 4: Table S3. The used antibodies [file 13578_2025_1392_MOESM4_ESM.docx]

| Supplementary Table 1 | | |
| --- | --- | --- |
| Reagents | Resource | Identifier |
| Antibodies | | |
| Rat monoclonal anti-BrdU | Abcam | Cat# ab6326 |
| Mouse monoclonal anti-nestin | BD Biosciences | Cat# 556309 |
| Mouse polyclonal anti-βIII -Tubulin（Tuj1） | Cell Signaling Technology | Cat# 2128 |
| Rabbit polyclonal anti-GFP | Proteintech | Cat# 50430-2-AP |
| Rabbit anti-Ki-67 | Millipore | Cat# AB9260 |
| Rabbit Polyclonal anti-Tbr2 | Abcam | Cat# ab23345 |
| Rabbit monoclonal anti-PAX6 | HuaBio | Cat# ET1612-89 |
| Mouse Monoclonal anti-GFAP | Cell Signaling Technology | Cat# 3670 |
| Mouse Monoclonal Anti-MAP2 | Sigma | Cat# M9942 |
| Mouse Polyclonal anti-Histone H3 | Abcam | Cat# ab1791 |
| Normal rabbit IgG antibody | Proteintech | Cat# B900610 |
| Rabbit anti-Zmynd11 | Novus | Cat# 39918 |
| Mouse anti-RNA polymerase 2 | Active Motif | Cat# 102660 |
| Rabbit anti-Epha2 | HUABIO | Cat# ER2001-31 |
| Rabbit anti-PDPK1 | HUABIO | Cat# ET1612-27 |
| Rabbit anti-Phospho-PDK1/PDPK1-S241 | Abclonal | Cat# AP0426 |
| Rabbit anti- Phospho-SGK1(Ser422) | Bioss | Cat# bs-3396R |
| Rabbit anti-SGK1 | Abclonal | Cat# A3936 |
| Rabbit anti-IRS1 | Abclonal | Cat# A0345 |
| Rabbit anti-Phospho-IRS1-S612/S636 | Abclonal | Cat# AP1507 |
| Mouse anti-Tubulin | HUABIO | Cat# M1305-2 |
| Mouse anti-GAPDH | Thermo Fisher Scientific | Cat# MA5-15738 |
| Rabbit anti-H3K36me3 | Active Motif | Cat# 61902 |
| Rabbit anti-PI3 Kinase p85 alpha | HUABIO | Cat# HA601206 |
| Rabbit anti-Phospho-PI3K p85(Y458)/p55(Y199) | Cell Signaling Technology | Cat# 4228S |
| Rabbit anti-SETD2 | Abclonal | Cat# A3194 |
| Rabbit anti-Zmynd11 | Abcam | Cat# ab190890 |
| Rabbit anti-H3K36me3 | PTMab | Cat# PTM-625 |
| Normal Rabbit IgG antibody | Proteintech | Cat# B900610 |
| AlexaFluor488 goat anti-rabbit | Thermo Fisher Scientific | Cat# A11008 |
| AlexaFluor488 goat anti-mouse | Thermo Fisher Scientific | Cat# A11001 |
| AlexaFluor568 goat anti-mouse | Thermo Fisher Scientific | Cat# A11004 |
| AlexaFluor568 goat anti-rabbit | Thermo Fisher Scientific | Cat# A11036 |
| AlexaFluor568 goat anti-rat | Thermo Fisher Scientific | Cat# A11077 |
